# Slug Is Increased in Vascular Remodeling and Induces a Smooth Muscle Cell Proliferative Phenotype

**DOI:** 10.1371/journal.pone.0159460

**Published:** 2016-07-21

**Authors:** Núria Coll-Bonfill, Victor I. Peinado, María V. Pisano, Marcelina Párrizas, Isabel Blanco, Maurits Evers, Julia C. Engelmann, Jessica García-Lucio, Olga Tura-Ceide, Gunter Meister, Joan Albert Barberà, Melina M. Musri

**Affiliations:** 1 Department of Pulmonary Medicine, Hospital Clínic-Institut d’Investigacions Biomèdiques August Pi i Sunyer (IDIBAPS), Universitat de Barcelona, Barcelona, Spain; 2 Biomedical Research Networking Center on Respiratory Diseases (CIBERES), Madrid, Spain; 3 Diabetes and Obesity Laboratory, IDIBAPS, CIBERDEM, Barcelona, Spain; 4 Institute of Functional Genomics, University of Regensburg, Regensburg, Germany; 5 Biochemistry Center Regensburg (BZR), Laboratory for RNA Biology, University of Regensburg, Regensburg, Germany; 6 Instituto de Investigación Médica Mercedes y Martín Ferreyra, INIMEC-CONICET, Universidad Nacional de Córdoba, Córdoba, Argentina; William Harvey Research Institute, Barts and The London School of Medicine and Dentistry, Queen Mary University of London, UNITED KINGDOM

## Abstract

**Objective:**

Previous studies have confirmed Slug as a key player in regulating phenotypic changes in several cell models, however, its role in smooth muscle cells (SMC) has never been assessed. The purpose of this study was to evaluate the expression of Slug during the phenotypic switch of SMC *in vitro* and throughout the development of vascular remodeling.

**Methods and Results:**

Slug expression was decreased during both cell-to-cell contact and TGFβ1 induced SMC differentiation. Tumor necrosis factor-α (TNFα), a known inductor of a proliferative/dedifferentiated SMC phenotype, induces the expression of Slug in SMC. Slug knockdown blocked TNFα-induced SMC phenotypic change and significantly reduced both SMC proliferation and migration, while its overexpression blocked the TGFβ1-induced SMC differentiation and induced proliferation and migration. Genome-wide transcriptomic analysis showed that in SMC, Slug knockdown induced changes mainly in genes related to proliferation and migration, indicating that Slug controls these processes in SMC. Notably, Slug expression was significantly up-regulated in lungs of mice using a model of pulmonary hypertension-related vascular remodeling. Highly remodeled human pulmonary arteries also showed an increase of Slug expression compared to less remodeled arteries.

**Conclusions:**

Slug emerges as a key transcription factor driving SMC towards a proliferative phenotype. The increased Slug expression observed *in vivo* in highly remodeled arteries of mice and human suggests a role of Slug in the pathogenesis of pulmonary vascular diseases.

## Introduction

In contrast to other terminally differentiated cells, differentiated/contractile SMC retain high plasticity and can undergo a phenotypic switch towards a synthetic/dedifferentiated state under specific stimuli [1]. This feature is common in vascular remodeling-associated diseases such as pulmonary hypertension (PH), chronic obstructive pulmonary disease (COPD), artheriosclerosis, aortic aneurysm and post-angioplasty restenosis [1–4], in which dedifferentiated SMC from the media translocate into the intima and proliferate [5–7]. The mechanisms mediating this phenomenon involve inflammation, shear stress, and hypoxia [4,8,9,10]. Dedifferentiated SMC that become proliferative and migratory, express more extracellular matrix components and fewer SMC contractile proteins [1,3].

Differentiation of SMC is regulated by transcriptional regulators of the myocardin-related transcription factor family (MRTF), such as myocardin and myocardin-like proteins 1 and 2 (MLK1 and MLK2) [11]. Myocardin (myoCD) increases the expression of actin cytoskeletal proteins via serum response factor (SRF). Conversely, SMC phenotypic switch is mediated by both the loss of positive differentiation signals and by the induction of multiple complementary repressor pathways, such as Krüppel-like factor 4 (KLF4) and transcription factor Sp1 (SP1) [12]. Interestingly, increased MLK1 expression has been involved in the phenotypic transition of endothelial cells (EC) in an *in vitro* model of endothelial injury [12]; and, SP1 appears to be the main transcriptional regulator of endothelial to mesenchymal transition (EnMT) in a model of inflammatory bowel disease [13,14], suggesting that transitional changes in cell phenotype involved in different clinical settings may be regulated by similar molecular pathways.

Slug belongs to the Snail transcription factor family that is involved in several biological functions, including epithelial to mesenchymal transition (EMT), cell differentiation, cell motility, cell cycle regulation, and apoptosis. Slug participates directly in the dissociation of cell-to-cell contacts by repressing endothelial cadherin (VE-cadherin) gene expression, and indirectly, by increasing extracellular matrix proteins [15,16]. Recently, the role of Slug in the control of different transcriptional programs of stem cell differentiation has been highlighted [17–20]; nevertheless its function in SMC has never been studied.

In the present study, we investigated the role of Slug in the phenotypic switch of SMC and its potential participation in the development of human pulmonary vascular remodeling. We showed that Slug induced SMC to undergo a proliferative phenotype by at least, modulating genes coding for cell proliferation and cell migration related pathways. Interestingly, we found increased levels of Slug, but not of Snail, in a mouse model of PH-related vascular remodeling and a positive correlation of Slug expression with the degree of both lung obstruction and arterial wall thickness. In addition, we observed Slug up-regulation in human pulmonary arteries with high degree of vascular remodeling. To our knowledge, this is the first time that Slug has been related to SMC proliferation and to vascular remodeling.

## Materials and Methods

### Primary cell cultures

Human pulmonary artery SMC were purchased from Lonza (Cologne, Germany). They were cultured with an appropriate growth medium, which consist in basal medium (SmBM), supplemented with growth factors (SingleQuot Kit Supplement; Lonza) and 10% fetal bovine serum (FBS) (Lonza) as previously performed [20]. All primary cultures were used at passages three to eight and were maintained in a humidified atmosphere at 37°C in 5% CO_2_.

### Cell models of *in vitro* differentiation

SMC differentiation was induced by cell-to-cell contact [21]. While cells grown at 70%–80% confluence (D0) comprised SMC with a dedifferentiated phenotype, cells grown at 100% confluence (D2) exhibited a more differentiated state. And, fully differentiated cells were obtained 4 days after reaching 100% confluence (D6). SMC differentiation under hypoxia was achieved by plating the cells at D0 or D6 in a hypoxic atmosphere of 1% O_2_ (*New Brunswick*^™^ *Galaxy*^®^ 170 R Incubator, Eppendorf, Hamburg, Germany) and harvested after four days of growth.

For *in vitro* studies with cigarette smoke extract (CSE), 50 ml of basal medium was bubbled with smoke of four unfiltered cigarettes (3RF, University of Kentucky, Lexington, KY), each containing 0.7 mg of nicotine and 9 mg of tar according to the manufacturer’s report, through a syringe-driven apparatus (Protowerx, Vanocuver, Canada). The CSE obtained was filtered through a 0.22 μm filter (Millipore, Bredfore, MA) and immediately frozen at -80°C until use. The pH of CSE was between 7.4 and 7.5 when diluted for each experiment. SMC at D6 were starved by washing three times with serum-free medium and incubated for 8 h to minimize the effects of FBS. Then, cells were incubated with diluted CSE (1/10) in starved medium for 24 h. Control cultures were treated with vehicle.

The SMC differentiation phenotype was determined by the relative expression of a smooth muscle contractile protein profile, which included myoCD, GATA-6, transgelin (sm22α), calponin and caldesmon, analyzed by real-time PCR (RT-PCR) and the decrease of the stem cell factor KLF4. The presence of filaments of smooth muscle α-actin (α-SMA), calponin and the smooth muscle specific myosin heavy chain (SM-MHC) were also assessed by immunofluorescence. The proliferative phenotype was evaluated indirectly by the expression of Ki67 by both RT-PCR and immunofluorescence and directly by cell cycle analysis. To determine cell cycle progression in SMC during differentiation, flow cytometry analysis was performed (Fortessa, Becton Dickinson, Franklin Lakes, NJ). Briefly, cells maintained in growth medium were permeabilized with ethanol 100% and incubated at -20°C during 30 min. Then, cells were incubated with 10 mg/ml of RNAsa A, and 1 mg/ml of propidium iodide for 30 min in dark at 37°.

We also assessed SMC differentiation in cells maintained at 70–80% confluence (D0) that were stimulated with transforming growth factor beta 1 (TGFβ1) (10 ng/mL; Acris, Herford, Germany) [22] for 24 h and 48 h in starved medium (SmBm supplemented with 1% inactive FBS). In this model, SMC differentiate before achieving 100% confluence. Dedifferentiation of SMC was induced in 100% confluent (D0) and in fully differentiated cells (D6) by incubation with the inflammatory cytokine TNFα (10 ng/ml; Bender Medsystems, Vienna, Austria) for 48 h in starved medium.

### Senescence experiments

Cellular senescence was evaluated with the Senescence Histochemical Staining Kit (Sigma-Aldrich, St Louis, MO) following the manufacturer’s guidelines. Dedifferentiated (passage 2) and fully differentiated SMC (passage 8) were compared to senescent SMC (passage 12). Briefly, cells were fixed using formaldehyde solution and incubated with X-gal. Pictures were taken after an ON incubation at pH 6. Blue senescent cells were counted and expressed as a percentage of the total number of nuclei DAPI-positive cells.

### RNA Isolation and Real Time PCR

Total RNA was isolated using TRIzol^®^ Reagent (Invitrogen) according to the manufacturer's instructions. Random-primed cDNA synthesis was performed at 37°C with 1 μg of RNA using the high capacity cDNA Kit (Applied Biosystems, Foster City, CA). Gene expression was measured by RT-PCR in a Chromo 4 Real Time PCR detector (Bio-Rad, Hercules, CA) and ABI fast 7900 HT (Applied Biosystems) using the sensiMix dt kit (Quantace, San Mateo, CA,) based on the DNA double-strand-specific SYBR green I dye and Taqman probes (Applied Biosystems) for detection. The results were normalized to GAPDH and β-actin expression levels and relative gene expression was analyzed by the 2-ddct method. The primers used and their sequences are listed in S1 Table.

### Western Blotting

SMC were washed with PBS and then lysated with RIPA buffer (50 mM TRIS-Cl, 150 mM NaCl, 1 mM EDTA, 10% NP-40, 0.10% deoxycholic acid) containing protease inhibitors (Sigma-Aldrich). The lysate was centrifuged at 14.000g for 15 min at 4°C to pellet debris and the protein-rich supernatant was stored at -20°C. Total protein concentration was measured with Bradford (Bio-Rad Laboratories, Hercules, CA). Mixed samples of three experiments per line containing 60 μg of protein were run in a SDS-PAGE on 5–12% Bis–Tris gel (Biorad) before transferring to a PVDF membrane. Blocking was done with 5% BSA (Sigma) and samples were incubated ON with primary antibodies. Primary antibodies against Slug (Cell Signalling, dilution 1/1000), and β-actin (Cell Signalling, dilution 1/1000) were revealed with HRP-labeled secondary antibody (Upstate, Charlottesville, VA). Blots were exposed with enhanced chemiluminescence (ECL) (Pierce, Thermo Scientific) and visualized in a LAS 4000 lumi-Imager (Bio-Rad). Membrane densitometry was analyzed with ImageJ software (Public domain). Blots were stripped using stripping buffer (50 mM Tris, 20% SDS and 0.7% ß-mercaptoethanol) and reprobed.

### Immunodetection

SMC differentiation was assessed by immunofluorescence using antibodies against α-SMA (1/750) and calponin (1/75) (DAKO Cytomation, Carpinteria, CA) and SM-MHC (1/250) (Abcam, Cambridge, UK). An antibody against antigen Ki-67 (1/50) (Novocastra^®^, Newcastle, UK) was used to measure cell proliferation. Briefly, cells were washed twice with PBS and fixed with 4% paraformaldehyde for 30 min. After permeabilization with PBS-0.1% triton, cells were washed and incubated with the appropriate antibody ON at 4°C. All primary antibodies were revealed with a secondary antibody conjugated with fluorescein during 90 min (Jackson Immuno Research, West Grove, PA) at room temperature. Nuclei were stained with DAPI. Immunofluorescence images were quantified by counting the number of immunoreactive cells with respect to the number of nuclei.

### Wound-healing assay

Forty thousand transfected cells were seeded in 48-well plates with growth medium. After 48 h of incubation the culture was scratched with a sterile tip and the medium was replaced for fresh growth medium. Pictures were taken at baseline and every 12 h. The healing area was analyzed with Image-Pro Plus software (Media Cybernetics, Inc.)

### Knockdown and overexpression experiments

To study Slug overexpression, 1 x 10^6^ cells were transfected with XL6 plasmid and XL6-Slug using the Amaxa Biosystem (Lonza) following manufacturer’s guidelines. One day after electroporation, cells were cultured with starved medium ON and stimulated with 10 ng/ml of TGFβ1 (Acris) for 24 h.

Slug knockdown was accomplished with 10nM of siPool against Slug (SiTool Biotech GmBH, Martinsried), which allows the use of low concentrations of siRNA minimizing off-targets effects [23]. A scrambled sequence was used as control. Both siPools were transfected using Lipofectamine RNAimax (Invitrogen).

### Transcriptome-wide gene expression analysis

SMC were transfected with 10 nM siSlug and mock-transfected in two biological replicates. RNA was isolated 48 h after transfection and further processed for transcriptome-wide expression profiles using the Human Gene 2.1 ST array platform (Affymetrix, Santa Clara, CA). Raw probe set intensities were processed and summarized using the robust multi-chip analysis (RMA) algorithm [24] with a custom chip definition file (CDF) from Brain array [25]. Expression was assessed for each gene based on linear modeling of the log2-normalized gene intensities using the R package Linear Models for Microarray Data [26]. Per-gene log2-fold changes and Benjamini–Hochberg false discovery rate (FDR)-adjusted p-values were estimated for Slug knockdown versus control. Slug knockdown samples in our array-based gene expression analysis, determined 355 (1200) genes that were differentially expressed with adjusted p-values of less than 0.01 (0.05).

### *In vivo* experiments

All procedures involving animals and their care were approved by the Ethics Committee of the University of Barcelona and the Institutional Committee of the University of Valladolid for Animal Care and Use. Furthermore, they were conducted according to institutional guidelines in compliance with national (Generalitat de Catalunya decree 214/1997, DOGC 2450) and international (Guide for the Care and Use of Laboratory Animals, National Institutes of Health, 85–23, 1985) laws and policies.

Our mouse model of pulmonary hypertension included 16 female mice (C57/bl6 mice around 22–25 g) that were divided in three groups. One group was maintained in normoxia and inoculated with vehicle (control, CTL; n = 6); the second group was exposed to chronic hypoxia using an initial ramp cycle to reach 10% O_2_, 5% CO_2_ for 3 weeks (chronic hypoxia, CH; n = 5); and the third group was exposed to CH and injected weekly with the VEGFR inhibitor, Sugen 5416 (Tocris, R&D Systems, Minneapolis, MN) at 20 mg/Kg (sub/cut) using a 25 gauge needle (CH+SU5416; n = 5) for three weeks. At the end of exposure, right ventricular pressure (RVP) was determined under anesthesia by inserting a pressure transducer (Milla,Houston, TX) into the right ventricle via the jugular vein. Animals were then sacrificed and their lungs processed for histology. The number of intrapulmonary vessels with a diameter <50 μm showing positive immunostaining for α-SMA were counted and expressed as a percentage of the total number of small vessels. The ventricle hypertrophy (Fulton index) was evaluated as the ratio between the right ventricle (RV) and left ventricle plus the septum weight.

### Pulmonary artery isolation

Segments of pulmonary artery were obtained from 19 surgical lung specimens that were resected for the treatment of localized lung carcinoma. The study was approved by the Ethics Committee of the Hospital Clinic, Barcelona, Spain. Arterial wall morphometry was analyzed in cross-sectional rings of pulmonary arteries after elastic orcein staining, as previously described [27]. Pulmonary arteries measuring approximately 2 cm long with an external diameter of 1–2 mm were dissected under microscope and cleaned of surrounding parenchyma and connective tissue. Segments were preserved in RNAlater^®^ solution (Ambion, Grand Island, NY) and frozen at -20°C until RNA extraction using TRIzol^®^ Reagent (Invitrogen, Grand island, NY). RNA quality was checked with the LabChip^®^ Test kit using the Agilent 2100 bioanalyzer (Agilent Technologies, Santa Clara, CA).

### Morphometry

The area of the intimal layer, expressed as a percentage of the cross sectional area of the pulmonary artery, was used to classify arteries into 3 groups by establishing the 33 and 66 percentiles as cut-off values. The different groups were identified as less remodeled (R1), mildly remodeled (R2) and highly remodeled (R3) pulmonary arteries.

### Immunohistochemistry

Slug expression was measured by immunohistochemistry in serial sections of highly remodeled (n = 3) and non-remodeled pulmonary arteries (n = 5). After hydration of the samples, bovine serum Albumin was used to block non-specific antibody interactions. The antibodies against Slug (1/75) (Cell Signaling, Boston, MA,) were added to the samples for 1 h at room temperature. After two rounds of washing, the secondary antibody was added for 45 min and contrasted with hematoxylin stain at room temperature. Areas positive for Slug in the arterial section were quantified with Image Pro and normalized to the total vessel wall area.

### Statistical analysis

All values are reported as mean ± SE. Determinations were performed in duplicate and at least 3 independent experiments were performed for each set of conditions. Two-group comparisons were analyzed using the two-tailed paired Student t-test for dependent samples or with the Mann–Whitney Rank Sum test for non-normally distributed data. Group comparisons were performed using one-way ANOVA or two-way ANOVA. Post hoc pairwise comparisons were made using the Student Newman–Keuls test for normally distributed variables or the Kruskal–Wallis and Dunn test for non-normally distributed variables. For all procedures, P-values lower than 0.05 were considered statistically significant.

## Results

### SMC phenotypic change *in vitro* models

**Fig 1:**
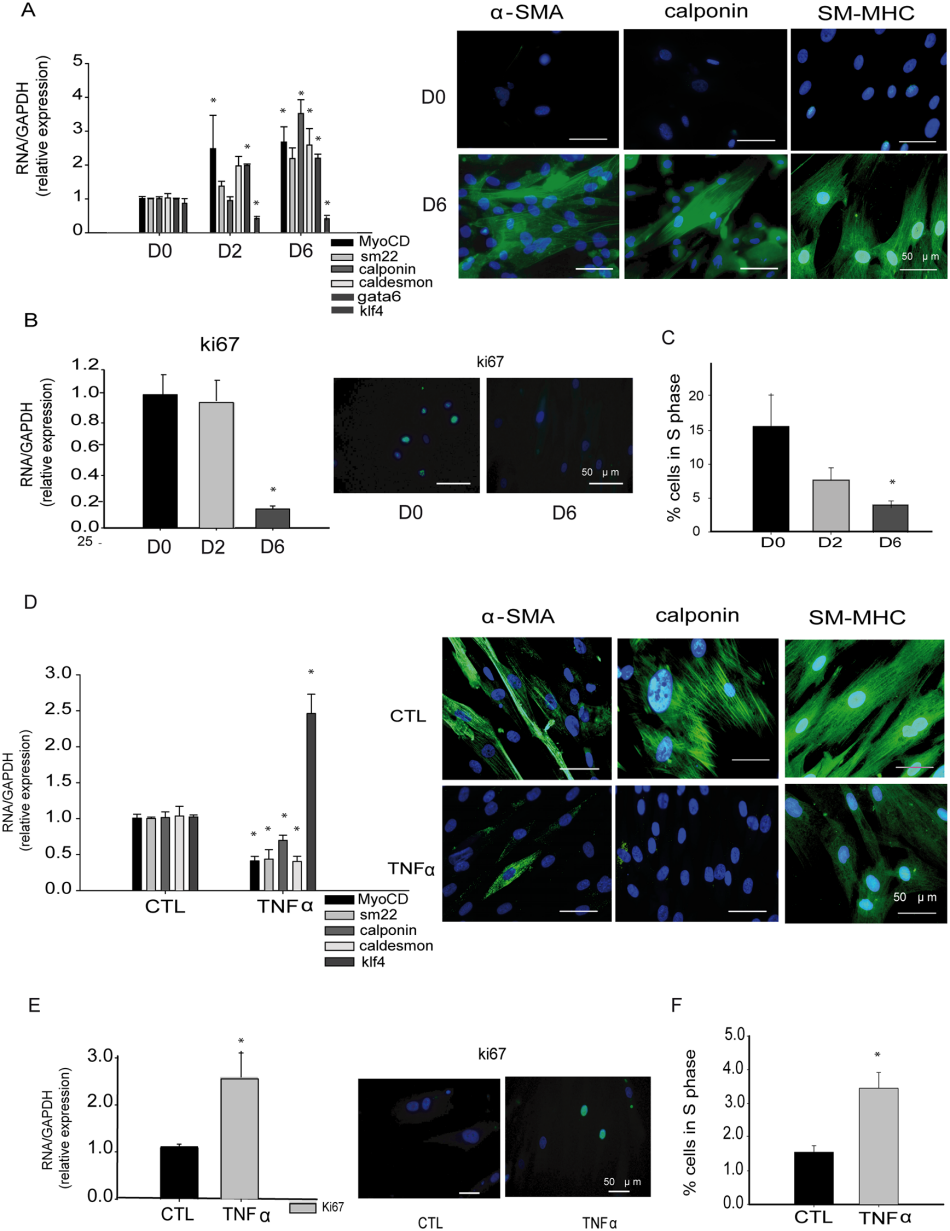
*In vitro* models of SMC phenotypic change. **A**, RT-PCR and immunofluorescence of the SMC differentiation markers (myoCD, sm22α, calponin, caldesmon, GATA6, α-SMA and SM-MHC, and the transcription factor KLF4 at D0, D2 and D6 states show the acquisition of a mature phenotype in SMC during differentiation. **B**, Cell proliferation decreases in differentiated cells, as determined by Ki-67 expression and **C**, cell cycle analysis. **D**, RT-PCR of SMC markers and KLF4 and immunofluorescence of α-SMA, calponin and SM-MHC in TNFα treated SMC showing the induction of SMC dedifferentiation by this cytokine. **E**, Gene expression of Ki-67 and **F**, cell cycle analysis increase significantly in TNFα treated SMC when compared with controls, indicating greater proliferation. Data are expressed as the mean ± SEM of five independent experiments performed in duplicate. *p < 0.05 by one-way ANOVA.

We analyzed the expression of contractile SMC markers in both cell-to-cell contact and TGFβ1 induced SMC differentiation and during TNFα-induced SMC dedifferentiation. Differentiated SMC phenotype induced by cell-to-cell contact was associated with higher expression of SMC contractile markers (*myoCD*, *gata-6*, *sm22α*, *calponin* and *caldesmon*) and decreased gene expression of the stem cell transcription factor *KLF4* (Fig 1A). Fully differentiated phenotype at D6 of culture was also confirmed by the strong expression of α-SMA, calponin and SM-MHC fibers, determined by immunofluorescence (Fig 1A). Similar results were obtained when differentiation was induced by 48 h of TGFβ1 treatment (S1 Fig). *Ki-67* expression (Fig 1B) and the number of cells in S Phase of the cell cycle, measured by flow cytometry (Fig 1C) decreased concomitantly with SMC differentiation indicating that SMC differentiation was concomitant with the decrease of cell proliferation. No changes in cell senescence were observed in highly confluent cells compared with proliferative cells (S2 Fig), indicating that cell senescence is not induced by cell confluence. Dedifferentiation of fully differentiated SMC was achieved by 48 h of treatment with the inflammatory cytokine TNFα, as shown by the decrease of contractile genes and an increase of *KLF4* expression (Fig 1D). α-SMA, calponin and SM-MHC fibers also decreased after 48 h of TNFα treatment (Fig 1D). Concordantly, increased proliferation was observed in TNFα-treated cells, as measured by both higher Ki-67 expression (Fig 1E) and increased cells in the S phase (Fig 1F).

### Slug expression correlated with a dedifferentiated phenotype

**Fig 2:**
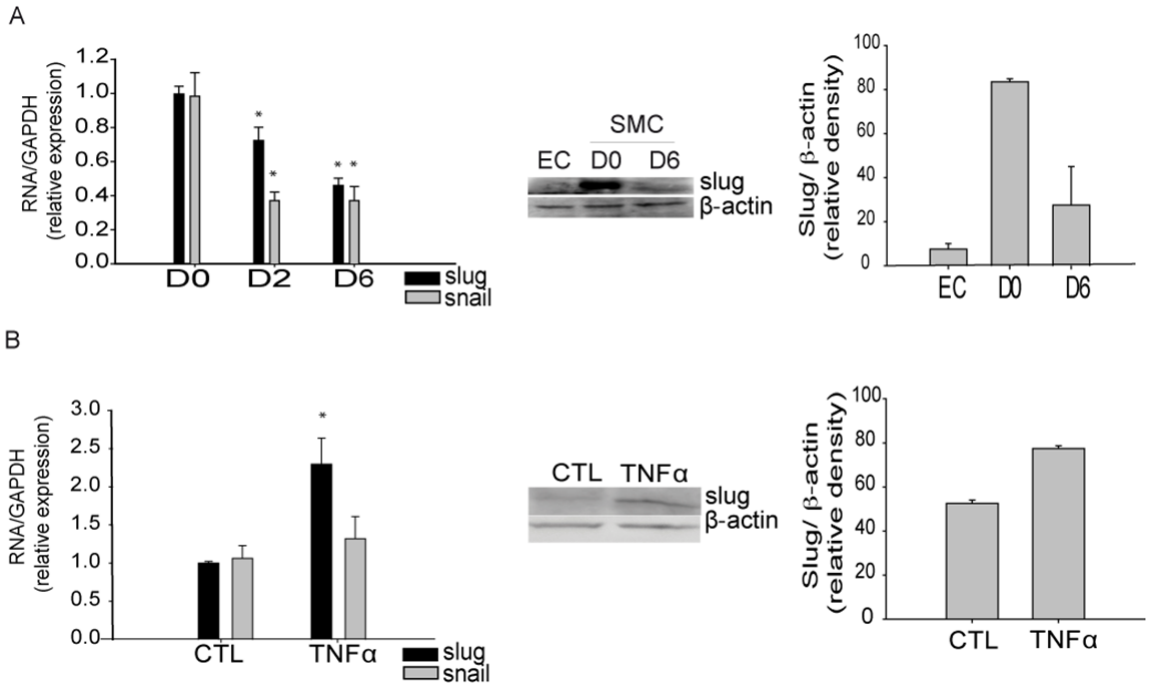
Slug is upregulated in dedifferentated SMC. **A**, Slug, and the related transcription factor Snail, decrease in mature SMC as determined by RT-PCR and western blot analysis. **B**, RT-PCR and western blot analyses show increased Slug expression in contractile SMC after 48 h of TNFα treatment. Data are expressed as the mean ± SEM of five independent experiments performed in duplicate. *p < 0.05 by one-way ANOVA.

We measured the expression of Slug and its related transcription factor Snail in these models. Expression of Slug and Snail were high in proliferative/dedifferentiated SMC (D0) and their expression decreased significantly to half in fully differentiated SMC (D6) in both cell-to-cell contact (Fig 2A) and TGFβ1-induced SMC differentiation (S1 Fig). Interestingly, induction of dedifferentiation by TNFα resulted in the increase of Slug expression but not of Snail expression (Fig 2B).

### Slug regulates proliferation and migration of SMC

**Fig 3:**
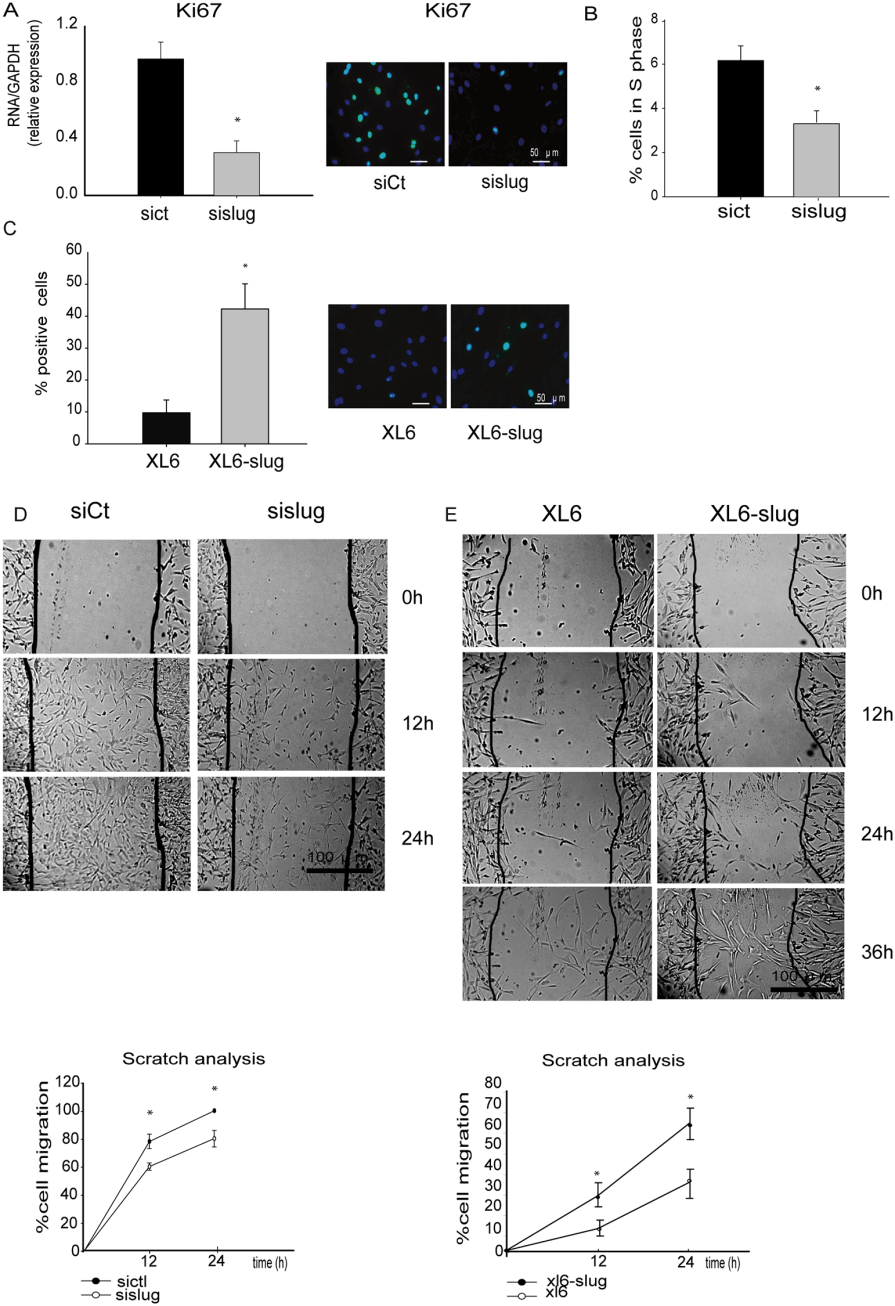
Slug regulates SMC proliferation and migration rate. **A,** RT-PCR and inmunofluorescence analysis showed that Slug inhibition promoted downregulation of Ki-67 expression, consistent with decreased S phase of cell cycle analyzed by flow cytometry (**B**). **C**, Slug overexpression increases SMC proliferation analyzed by Ki67 expression. **F** and **G,** Cell migration was measured by wound-healing assay and expressed as the percentage (%) of SMC migrating/time. Slug knockdown cells display lower migration rates than control cells (F), while Slug-overexpressing SMC exhibit higher migration rate than control cells (G). Data are expressed as the mean ± SEM of four independent experiments performed in duplicate. *p < 0.05 by paired *t*-test.

In order to investigate whether Slug expression is associated with changes in the proliferative/migrative phenotype of SMC, gain and loss of function of Slug were performed. Knockdown of Slug was achieved by using a SiPool against Slug, which resulted in the 90% of Slug knockdown (S3 Fig). Slug knockdown in highly proliferative cells resulted in a significant decrease in both *Ki-67* expression (Fig 3A) and cells in S phase (Fig 3B). By contrast, Slug overexpression increased the number of proliferating cells (Fig 3C). These results indicate that Slug expression sustain SMC proliferation. Migration, measured as the capacity of cells to resolve an *in vitro* wound was also measured (Scratch assay). Slug knockdown resulted in the reduction of migrating cells (Fig 3D), whereas Slug overexpression was associated with an increase in the migration rate of SMC (Fig 3E), indicating that Slug stimulates a migratory phenotype in SMC.

### Slug regulates both TNF-α and TGFβ1-induced SMC phenotypic change

**Fig 4:**
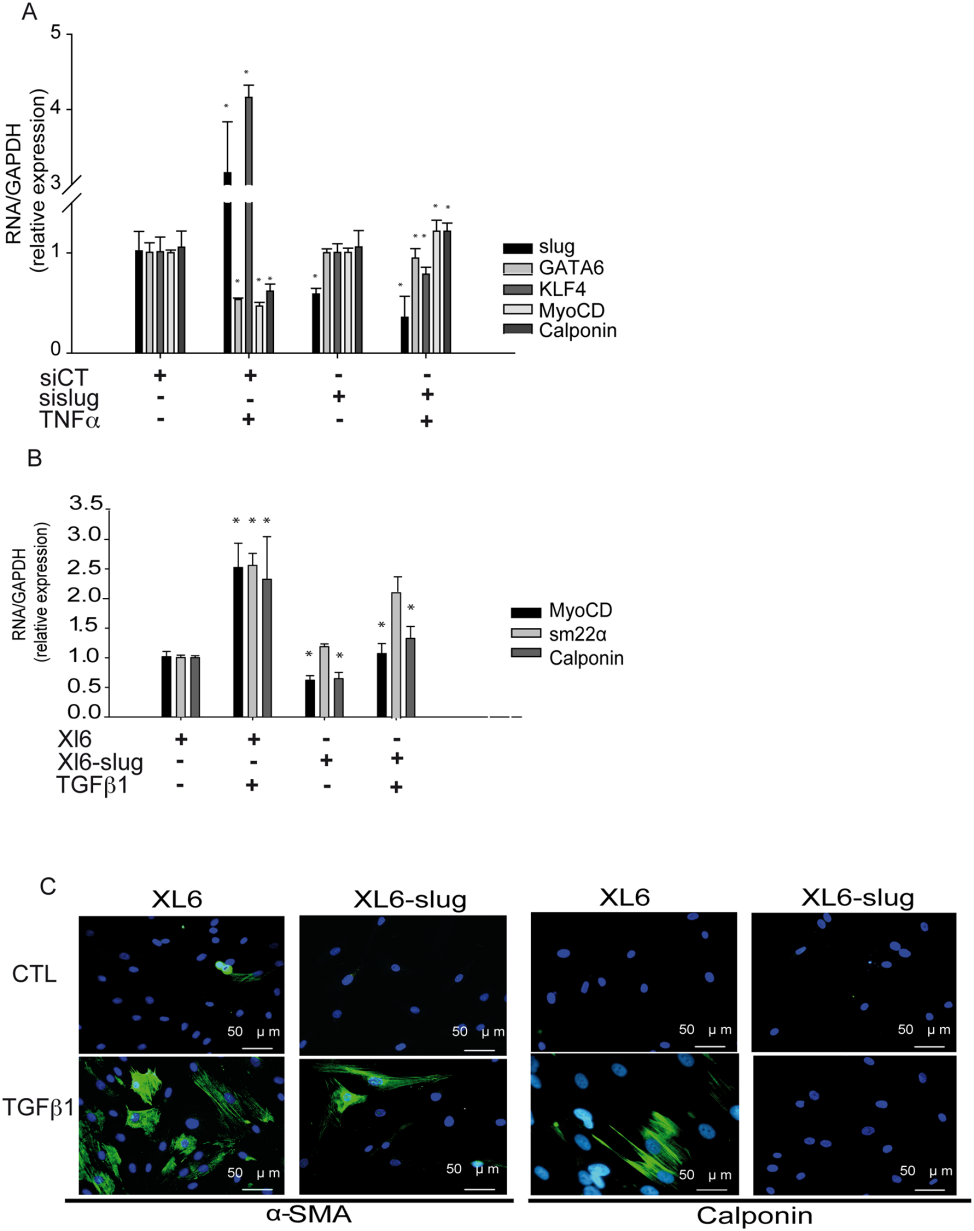
Slug regulates both TNFα-induced dedifferentiation and TGFβ1-induced differentiation. **A,** The increase of KLF4 and the decrease of SMC marker genes induced by TNFα treatment is prevented by Slug knockdown measured by RT-PCR **B-C,** TGFβ1-induced increase of SMC markers is dampened in Slug overexpressing cells as observed by RT-PCR (B) and immunofluorescence (C). Data are expressed as mean ± SEM of three independent experiments performed in duplicate. *p < 0.05 by paired *t*-test.

We reasoned that if Slug induces proliferation and migration in SMC, it could also regulate changes in SMC phenotypic switch. Slug knockdown prevented the TNFα-induced dedifferentiation of SMC denoted by the restored expression of *myoCD*, *gata-6*, *calponin* and KLF4 following TNFα treatment (Fig 4A). Transient overexpression of Slug disturbed the TGFβ1-induced SMC differentiation, which resulted in a lower expression of both myoCD and calponin after 48 h of treatment and a decreased α-SMA and calponin fibers (Fig 4B and 4C).

### Slug regulates genes related to cell migration and proliferation in SMC

**Fig 5:**
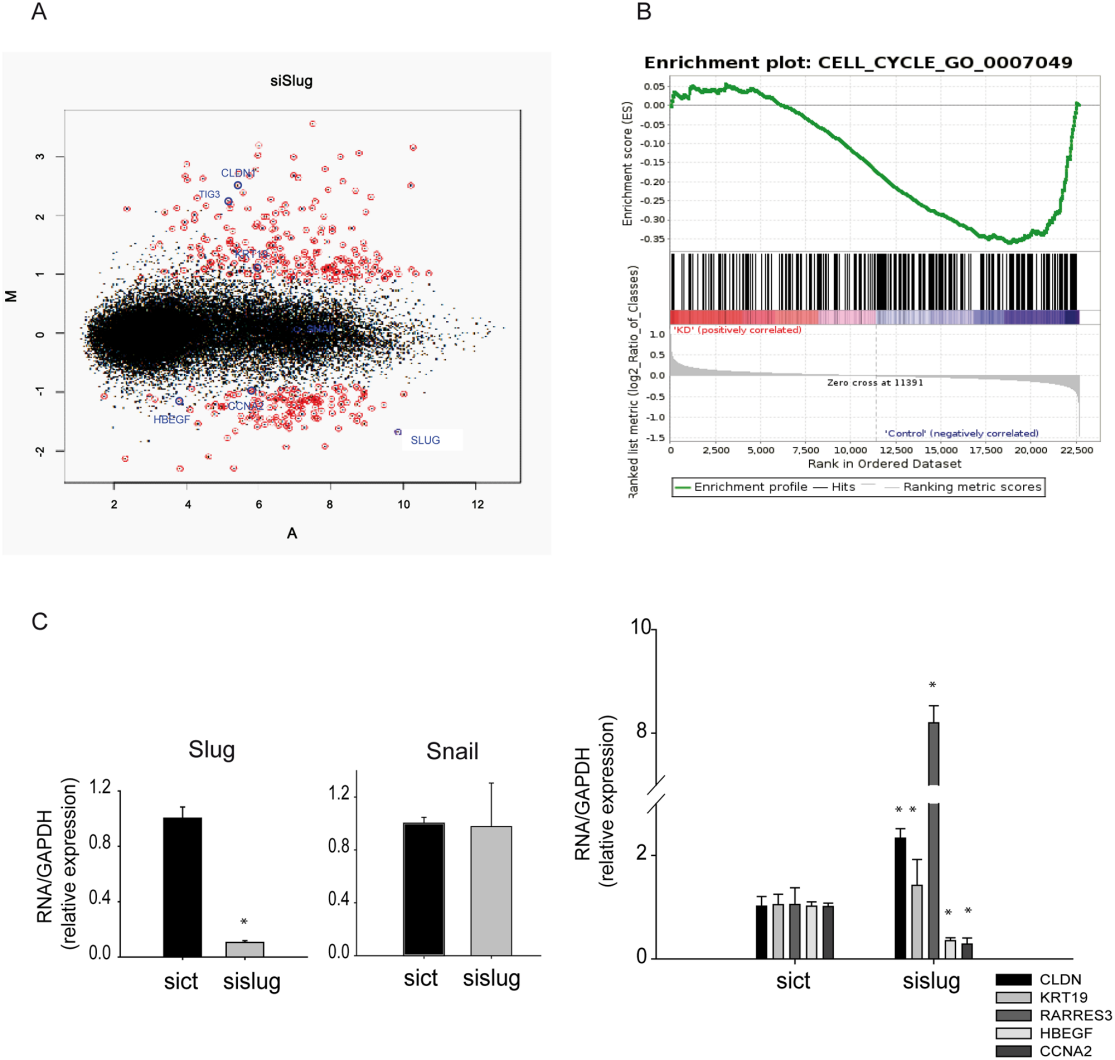
Slug regulates genes related to proliferation and migration pathways. **A,** Scatter plot of the per-gene log2-fold changes following Slug knockdown (x-axis). Differentially expressed genes in Slug knockdown cells respect to control are marked in red. Validated genes are marked in blue. The vertical dashed lines mark the two fold changes. **B,** Enrichment plot of the GSEA cell cycle. GSEA gave a normalized enrichment score of -1.621 and an FDR of 0.0, indicating a significant enrichment of downregulated cell cycle-associated genes. **C**, Validation of Slug, Snail and other genes array performed by RT-PCR show the downregulation of HBEGF, CCNA2, and the upregulation of CLDN1, RARRES3 and KRT19. Data are expressed as the mean ± SEM of five independent experiments performed in duplicate. *p < 0.05 by one-way ANOVA.

Differential gene expression analyses using Affymetrix Human Gene 2.1 ST arrays were performed 48 h after Slug knockdown in SMC (Fig 5A and S1 Appendix). GO terms analysis of Biological Processes that are modified after Slug knockdown revealed that cell proliferation was one of the most altered pathways. In addition, GSEA analysis showed an enrichment of downregulated genes related to proliferation (Fig 5B and S3 Table). We validated the up-regulation of the tumor suppressor retinoic acid receptor responder protein 3 (RARRES3, also known as TIG3) after Slug knockdown (Fig 5C). Furthermore, the decrease of two pro-proliferative related genes, Cyclin A2 (CCNA2) and Heparin-Binding Epidermal Growth Factor (HBEGF), after Slug inhibition are shown in Fig 5C. Increased levels of two known Slug targets, claudin1 (CLDNI) and keratin19 (KRT19) were also validated (Fig 5C). RARRES3, CLDNI and KRT19 increased along differentiation, whereas CCNA2 and HBEGF decreased (S4 Fig). These results correlate well with our findings in siSlug-transfected cells.

### Slug is increased in mice with severe pulmonary hypertension

**Fig 6:**
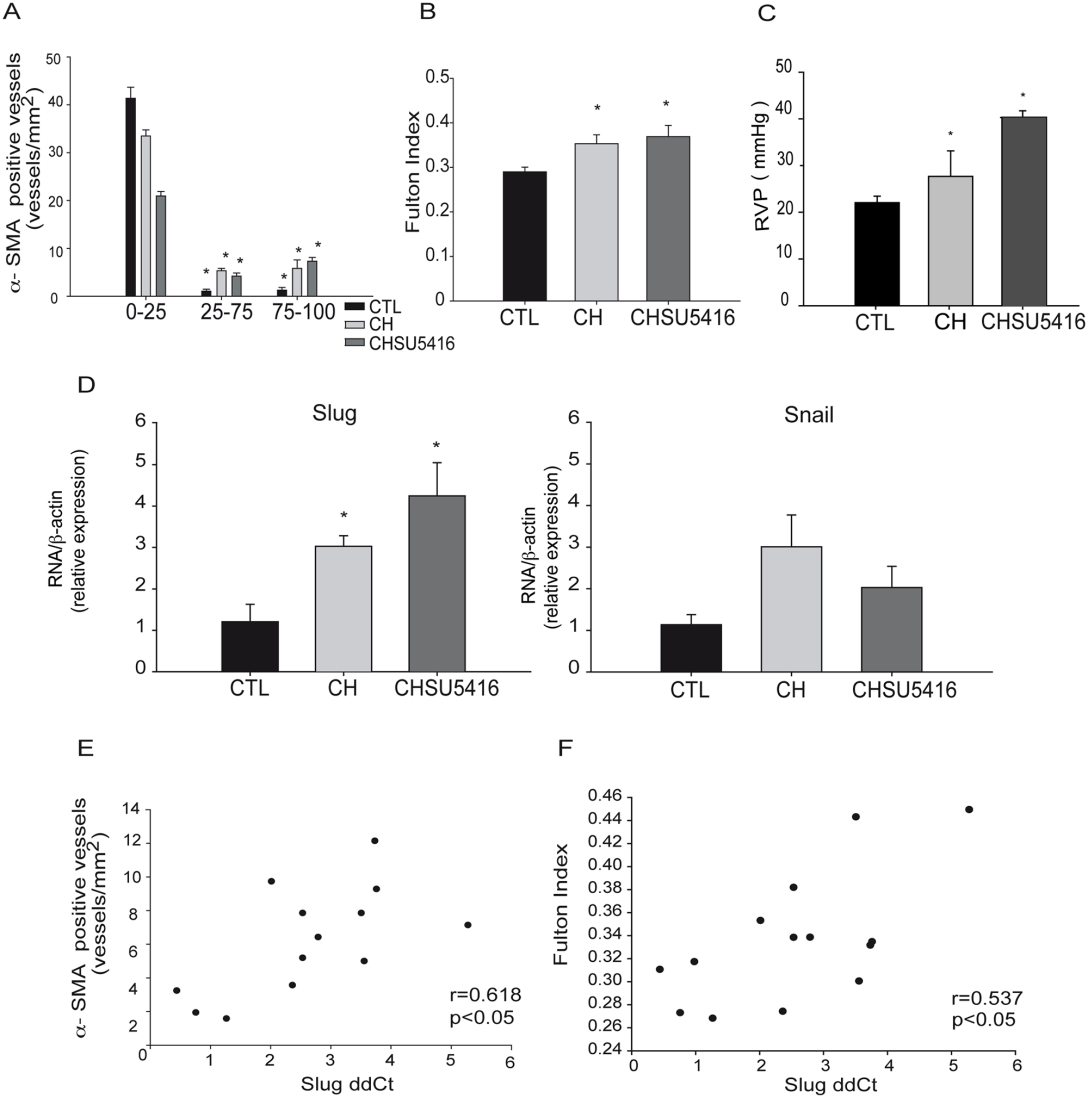
Analysis of Slug expression in the lungs of a mouse model of severe PAH. **A**, Partially (within 25–75% visible muscularization) and totally muscularized (within 75–100% visible muscularization) intrapulmonary vessels with a diameter <50 μm analyzed in CTL: control group (n = 6), CH: animals exposed to chronic hypoxia (n = 5), CH+SU5416: animals exposed to chronic hypoxia plus Sugen 5416 (n = 5) show positive immunostaining for α-SMA in CH and CH+SU5416 animals. *p<0.05 by one way ANOVA. **B-C**, Fulton index (B) and Right ventricular pressure (RVP) (C) are increased in CH and CHSU5416. **D**, RT-PCR in lung homogenates showed increased Slug, but not Snail expression, in the group exposed to CH and CHSU5416 analyzed by one-way ANOVA. **E**, Correlation between Slug expression (1/dCt) with both the number of α-SMA positive vessels and **F**, the Fulton index (note that the lower the 1/dCt, the higher the Slug expression level). *p < 0.05 by Spearman analysis.

Treatment of mice with chronic hypoxia (CH) in combination with the VEGFR inhibitor, SU5416 (CH+SU5416), reproduces the severe PAH observed in humans. As described previously [28], muscularization of vessels was increased in both CH and CH+SU5416, with respect to control mice (Fig 6A). RV hypertrophy was confirmed by the increase in the Fulton Index in both CH and CH+SU5416 (Fig 6B). Right ventricular pressure (RVP) was increased in animals exposed to CH or CH+SU5416 (Fig 6C). RT-PCR analysis in lung homogenates showed a moderate increase of Slug expression in CH and CH+SU5416 animals (Fig 6D). There were no statistically significant changes in the gene expression of Snail in CH animals compared with control mice (Fig 6D). A positive correlation was found between Slug expression and both the number of vessels positive for α-SMA and the Fulton Index (Fig 6E and 6F). Accordingly, *in vitro* treatment *of* SMC for 24 h with CH or cigarette smoke extract (CSE), two well-known inducers of vascular remodeling, stimulated both the increase of Slug and SMC phenotypic switch (S5 Fig).

### Expression of Slug is enhanced in highly remodeled human pulmonary arteries

**Fig 7:**
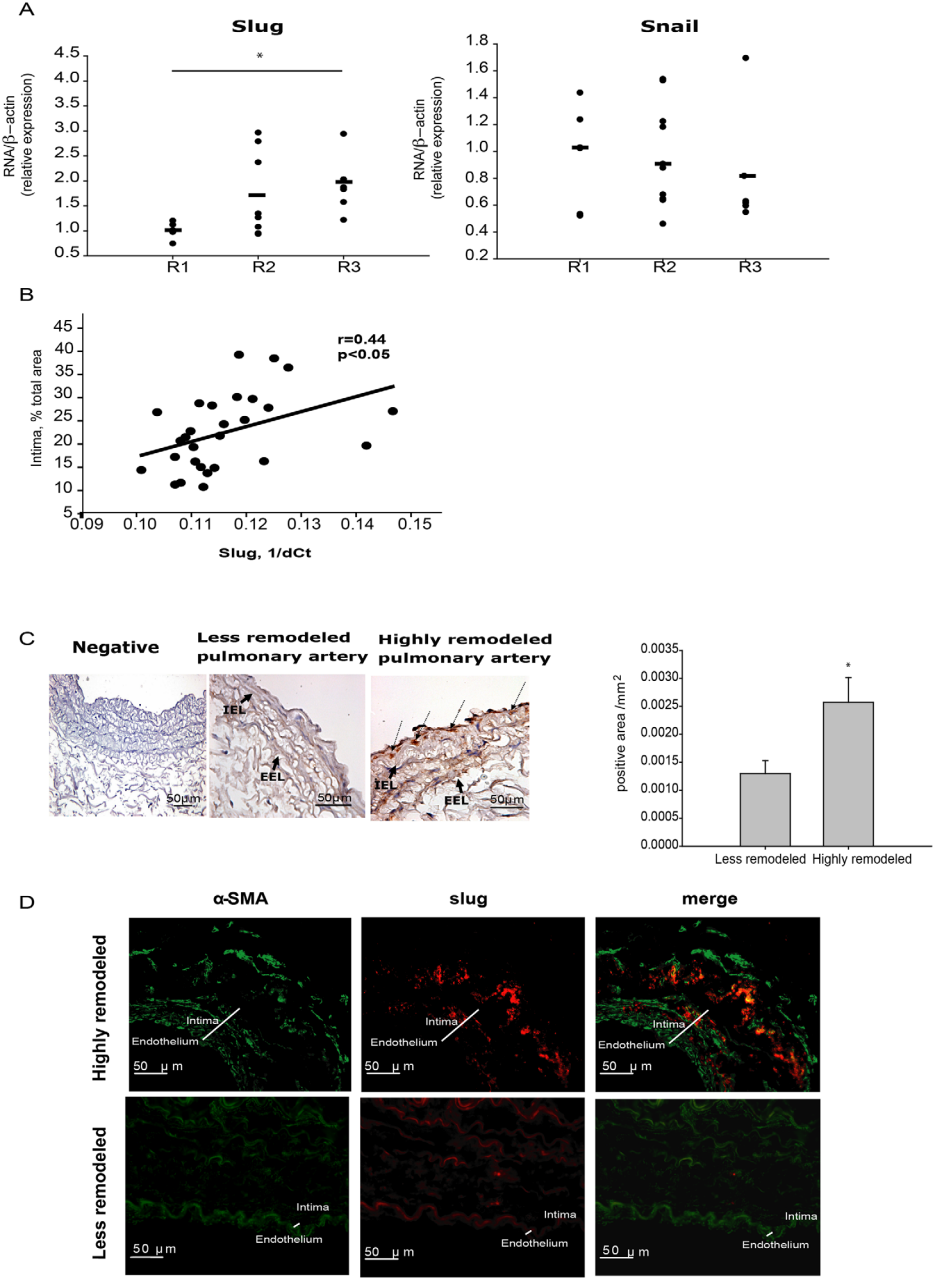
Analysis of Slug expression in human pulmonary arteries. **A,** Samples were classified according to the degree of vascular remodeling: low (R1), mild (R2) and high (R3). Slug but not Snail expression was significantly increased in R3 arteries compared with R1. *p<0.05 by one way ANOVA. **B**, Slug expression (1/dCt) displays a positive correlation with the intimal enlargement of pulmonary arteries measured by % of intimal thickness of the pulmonary artery (r = 0.44, p<0.05 by Pearson test). Note that the lower the 1/dCt, the higher the expression level. **C**, Representative images of Slug immunostaining; Slug expression is significantly increased in dedifferentiated intimal pulmonary SMC and EC of highly remodeled pulmonary arteries (right image) compared with less remodeled (left image). Negative control is shown in the first picture. Slug positive areas were quantified in highly remodeled (n = 3) and less remodeled (n = 4) arteries and normalized by the total wall area. Highly remodeled pulmonary arteries display a significant increase in Slug positive cells compared with less remodeled arteries. *p<0.05 by *t*-test. **D,** Slug (red), α-SMA (green) and merged (yellow) immunofluorescence images. Slug expression is predominantly found in the intima layer of remodeled pulmonary arteries.

Next, we also tested whether or not Slug expression was altered in human during vascular remodeling. Seven patients with COPD and eleven subjects with normal lung function (four non-smokers and seven smokers) were enrolled in our study (S4 Table). Patients with COPD had significantly lower forced expiratory volume in the first second (FEV_1_), that is the most frequent index for assessing airway obstruction; lower FEV_1_/Forced vital capacity (FVC) ratio, which indicate increased airway resistance to expiratory flow; lower diffusing capacity for carbon monoxide (DLCO), which measures the ability of the lungs to transfer gas inhaled air to the red blood cells in pulmonary capillaries, and mild hypoxemia, than healthy controls. Groups formed according to their degree of remodeling were matched for subjects with COPD and subjects with normal lung function; and, there were no differences in respiratory variables between groups. RT-PCR analysis showed that expression of Slug, but not of Snail, was significantly upregulated in highly remodeled pulmonary arteries (R3) compared to less remodeled arteries (R1) (Fig 7A). Additionally, a positive correlation between Slug gene expression and the thickness of the intima layer was found (Fig 7B). Slug localization in cross-sectional artery rings was assessed by immunohistochemistry and co-immunofluorescence with α-SMA antibody. Slug was barely detected in the intimal layer of less remodeled arteries (Fig 7C). In contrast, highly remodeled arteries showed strong intensity in the intima and media layer (Fig 7D).

## Discussion

Although many reports have described key factors involved in the regulation of the SMC phenotypic switch [3], the detailed molecular mechanisms driving this process are not yet fully understood. Furthermore, many of the described players *in vitro* have not been found to be associated with vascular disease. In this study, we show that the EMT-related transcription factor Slug is highly expressed in proliferative SMC, and its expression decreases significantly as maturity increases. Diminished Slug expression, both during differentiation and after knocking down its expression, correlated with a reduced proliferation, therefore, suggesting that Slug regulates the expression of genes related to these pathways. Our genome-wide transcriptome study revealed that Slug modulates the expression of many genes that regulate proliferation, which is consistent with cell cycle assays. Specifically, downregulation of Slug in SMC was related with a decreased expression of both HBEGF and CCNA2, whereas the genes CLDNI, KRT19 and RARRES3 increased their expression. HBEGF is a stimulator of SMC proliferation and it is known to play an important role in neointimal formation of a rat model [29]. The CCNA2 gene encodes for cyclin-A2, a protein involved in cell cycle progression. Nevertheless Slug is a transcriptional repressor. And, the expression of both the CCNA2 and HBEGF genes decreased during its knockdown, suggesting that they are indirect targets of Slug. Downregulation of Slug in SMC was related with a greater expression of two known Slug target genes, CLDNI and KRT19. These genes are involved in adhesion/migration pathways [30,31], which may explain the increased rate of migration observed following Slug overexpression. Interestingly, both genes are described as specific epithelial genes [30,31]. However, we observed expression of these genes in differentiated SMC cells and in Slug-knocked down SMC, indicating that they also play a role in this cell type. Expression of KRT19 has been also previously described in vascular smooth muscle and skeletal muscle [32,33] RARRES3 is a tumor suppressor [34] gene that maintains cell adhesion and decrease metastasis [35]. Concordantly, its expression was increased after Slug knockdown. Overall, these results indicate the important physiological role of Slug in cell proliferation and migration in SMC.

Our study revealed that Slug induces a proliferative/dedifferentiated phenotype in SMC. This was demonstrated in the Slug overexpression experiments. In these assays, Slug promoted an increased rate of proliferation and migration and blocked the TGFβ1-induced SMC differentiation. These results agreed with a number of previous works demonstrating the key role of Slug in regulating cell dedifferentiation, proliferation and migration. Slug upregulation was found to confer a stem cell-like phenotype in a breast cancer model and in epithelial corneal cells [36,37]. Recently, it was also shown that Slug blocked differentiation of both striated muscle cells and epidermal cells [17,18]. Slug is a zinc finger transcription factor that suppresses gene expression through binding to E-boxes in the promoter of its target genes [16, 38]. In striaded muscle cells, Slug represses differentiation by competing with the bHLH transcription factor MyoD, which is the main transcription factor driving differentiation of these cells, for binding to the E-boxes of specific muscle genes [18]. Many smooth muscle-specific genes, including α-SMA, sm22α and SM-MHC, contain E-boxes in their promoters and are activated by class I bHLH transcription factors [39]. Also, twist1, a known inductor of EMT, has also been shown to bind to E-boxes in the promoter region of both sm22α [40] and α-SMA, and repress them *in vitro* and *in vivo* [40]. In our work, we observed a lack of activation of sm22α and α-SMA, following Slug knockdown in proliferative cells (S1 Appendix), demonstrating that Slug is not directly repressing them at this stage of differentiation. However, it cannot be discarded that Slug displaces bHLH transcriptional activators once these genes are activated in differentiated cells.

**Fig 8:**
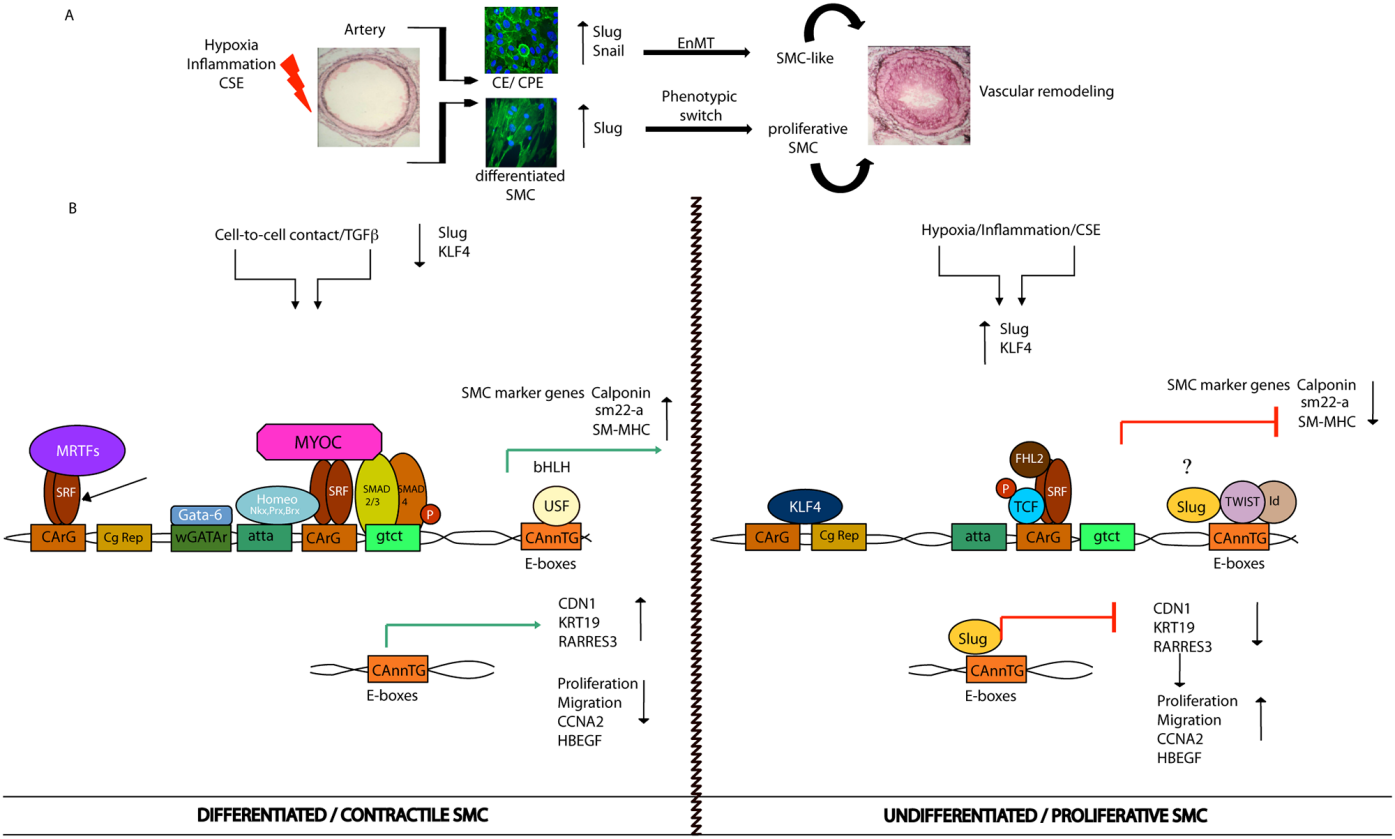
Slug induces a proliferative phenotype of SMC. **A,** Schematic figure showing the contribution of both Slug and Snail to the phenotypic switch of EC and SMC. Under an inflammatory/hypoxic environment the expression of Slug and Snail increase in EC and promotes EnMT, while only Slug increases in SMC to induce a proliferative SMC phenotype. **B,** Schematic figure representing the most prominent molecular players involved in the phenotypic switch of SMC. In contractile cells, MYCD-SRF/MRTF-SRF complexes bind to CArG boxes, while bHLH transcription factors, like USF bind to E-boxes to activate and maintain expression of SMC specific markers. In this stage, Slug is minimally expressed. In consequence, expression of its known targets, like CDN1 and KRT19, as well as the tumor suppressor RARRES3, are high and migration is repressed. Expression of the cell cycle related genes, CCNA2 and HBEGF, are highly expressed and the rate of proliferation is very low. Under specific stimuli, such as inflammation and/or hypoxia, the expression of Slug is triggered. Expression of its target genes CDN1 and KRT19 are blocked and cell migration is activated. Moreover, expression of CCNA2 and HBEGF decrease and cells become proliferative. In this condition, the expression of the stem cell factor KLF4 is upregulated. Expression of MYCD and its binding to CArG boxes is also repressed resulting in decreased expression of SMC specific markers. MYCD, myocardin; SRF, serum response factor; MRTF, myocardin-related transcription factors; KLF4, krüppel like factor 4; CDN1, claudin1; KRT19, keratin 19; RARRES3, retinoic acid receptor related gene 3; CCNA2, cyclinA2; HBEGF, Heparin-Binding EGF-Like Growth Factor.

TNFα treatment to induce a phenotypic switch from fully differentiated SMC was used in this study. Elevated levels of TNFα, or systemic inflammation, have been associated with blood vessel remodeling [41,42]. Specifically, TNFα is thought to be involved in several chronic hypoxia-associated lung diseases like COPD [43,44]. In our model, TNFα induced Slug but not Snail up-regulation, and induced contractile protein downregulation in differentiated cells with a concomitant increase of a migrative/proliferative phenotype. These results correlate well with previous work where TNFα inhibited the contractile phenotype of cerebral vascular SMC, inducing pro-inflammatory/matrix-remodeling genes [45], and stimulated Slug expression in breast cancer cells [46]. We observed that preventing the upregulation of Slug by transfection of the siSlug, following TNFα treatment, blocked the inflammatory-induced SMC phenotypic switch. Interestingly, Slug knockdown was able to prevent the TNFα-induced up-regulation of KLF4, which is a known inductor of SMC dedifferentiation [47]. We also observed increased Slug, but not Snail expression, in lungs of mice during vascular remodeling and in highly remodeled human pulmonary artery samples. This interesting asymmetry observed in the behavior of Slug and Snail has been previously described in several studies. Shirley and colleagues described that the two factors are not induced by identical stimuli [48]. In addition, Slug, but not Snail mRNA was elevated during wound reepithelialization *in vitro and*, *ex vivo* and *in vivo* in mice [49]. Moreover, to induce EMT, Slug and Snail showed overlapping, as well as a range of other functions. Slug seems to act early to trigger EMT, whereas Snail acts later to complete the process [50,51]. Remarkably, Snail knockout mice died at gastrulation [52], while Slug knockout mice were viable [53]. Altogether, these studies clearly show that Slug and Snail are not functionally equivalent, although they share certain targets in cell differentiation processes. Interestingly, a significant number of Slug overexpressing mice died of cardiac hypertrophy and cardiac failure exhibiting the key role of this factor in the cardiovascular system homeostasis. In correlation with these observations, using a murine model of severe pulmonary hypertension induced by CH, we found greater expression of Slug in lung homogenates of CH-exposed animals, as well as a correlation of Slug with the severity of vessel remodeling. These findings support a stimulating role of Slug in hypoxia-induced SMC proliferation. In correlation, *in vitro* treatment of SMC with CH or CSE, both of which are reported to regulate cell proliferation during vascular remodeling [54,55], induced the expression of Slug concomitantly with cell differentiation defects. Interestingly, we also found a correlation between the intima thickness of human pulmonary arteries and Slug expression. A recent report from Owens and colleagues, using a cell-tracing system, showed that more than 80% of medial SMC during intima hyperplasia in mice undergo phenotypic switch [47]. Also, EnMT, a process in which both Slug and Snail are involved, was also observed *in vivo* during the PH-related vascular remodeling (Fig 8A) [56]. In this work, we documented that highly remodeled arteries showed a strong intensity of Slug, but not of Snail, in the intima and media layer, indicating that a greater number of SMC-like cells in the intima could arise from the media through phenotypic switch of SMC, likely driven by Slug (Fig 8A).

In summary, Slug is revealed as a key transcription factor driving a proliferative/migratory phenotype of SMC. Our results indicate that in an inflammatory environment, the expression of Slug is induced in SMC, which in turn, stimulates a SMC proliferative/dedifferentiated phenotype, at least through modulating proliferation and migration genes (Fig 8B). To the best of our knowledge, this is the first time that Slug has been studied during the phenotypic switch of SMC. The increased expression of Slug observed in remodeled human pulmonary arteries, suggests that this transcription factor has an important role in the pathogenesis of pulmonary vascular impairment that is promoted by an inflammatory and/or hypoxic environment. Accordingly, therapies targeting Slug could be of potential interest in the treatment of pulmonary vascular disorders.

## Supporting Information

S1 Appendix Summary Table of the gene expression in SMC after 48h of Slug knockdown. (XLSX)

S1 Fig SMC differentiation model induced by TGFβ1 treatment. **A**, RT-PCR and **B**, immunofluorescence of SMC markers show SMC differentiation after 48 h of TGFβ1 treatment. Slug expression, measured by RT-PCR, decreases in differentiated cells.
(PDF)

S2 Fig Analysis of cell senescence in the cell-to-cell contact model. Graph showing the percentage of senescent cells expressed as b-galactosidase positive cells/total cells. No differences are observed between D0 and D6 (passage 2–8). Passage 12 is used as a control for cell senescence. *p < 0.05 by one-way ANOVA
(PDF)

S3 Fig Slug siRNA Knockdown efficiency. Representative Slug immunofluorescence, which demonstrates the decrease of Slug expression around 90% with respect to control cells.
(PDF)

S4 Fig Gene expression of selected genes from the microarray, during SMC differentiation. KRT19 (A), RARRES3 (D) and CLDNI (E) increase in differentiated SMC, whereas CCNA2 (C) and HBGEF (B) decrease.
(PDF)

S5 Fig Analysis of Slug in SMC subjected to hypoxia and CSE. **A-B** Slug expression increases in SMC, correlating with SMC dedifferentiation, as shown by the downregulation of SMC marker genes, in hypoxia-induced cells. **C,** CSE stimulation promotes the increase of Slug expression and the decrease of SMC differentiation markers. Data are expressed as the mean ± SEM of at least three independent experiments performed in duplicate. *p < 0.05 by paired *t*-test.
(PDF)

S1 Table Primers. (PDF)

S2 Table Summary of the top 15 GO terms analysis of Biological pathways. (PDF)

S3 Table Summary of the top 30 genes contributing to the enrichment of downregulated cell cycle genes. (PDF)

S4 Table General characteristics of the population. * p<0.005 vs NS. ^†^ p<0.005 vs S.
(PDF)

## Abbreviations

SMC: Smooth Muscle Cells
EC: Endothelial Cells
EnMT: Endothelial to Mesenchymal Transition
MyoCD: Myocardin
SM-MHC: smooth muscle-myosin heavy chain 11
KLF4: Kruppel-like Factor 4
Sm22α: Transgelin
α-SMA: α-Smooth Muscle Actin
R1: less remodeled arteries
R2: mildly remodeled arteries
R3: highly remodeled arteries
TNFα: Tumor Necrosis Factor alpha
TGFβ1: Transforming Growth Factor beta-1
VEGFR: Vascular Endothelial Growth Factor Receptor
RARRES3: Retinoic Acid Receptor Responder Protein 3
HBEGF: Heparin-Binding Epidermal Growth Factor
KRT19: Keratin 19
CLDN1: Claudin 1
CNNA2: Cyclin A2
CH: Chronic Hypoxia
CS: Cigarette Smoke

## References

[1] Owens GK. Regulation of differentiation of vascular smooth muscle cells. Physiol Rev 1995;75:487–517. 7624392 10.1152/physrev.1995.75.3.487

[2] Ailawadi G, Moehle CW, Pei H, Walton SP, Yang Z, Kron IL, et al Smooth muscle phenotypic modulation is an early event in aortic aneurysms. J Thorac Cardiovasc Surg 2009;138:1392–1399. 10.1016/j.jtcvs.2009.07.075 19931668 PMC2956879

[3] Owens GK. Molecular control of vascular smooth muscle cell differentiation and phenotypic plasticity. Novartis Found Symp 2007;283:174–191. 18300422 10.1002/9780470319413.ch14

[4] Ross R. Atherosclerosis is an inflammatory disease. Am Heart J 1999;138:S419–S420. 10539839 10.1016/s0002-8703(99)70266-8

[5] Stenmark KR, Davie N, Frid M, Gerasimovskaya E, Das M. Role of the adventitia in pulmonary vascular remodeling. Physiology (Bethesda) 2006;21:134–145. 16565479 10.1152/physiol.00053.2005

[6] Stenmark KR, Fagan KA, Frid MG. Hypoxia-induced pulmonary vascular remodeling: cellular and molecular mechanisms. Circ Res 2006;99:675–691. 17008597 10.1161/01.RES.0000243584.45145.3f

[7] Tanaka K, Sata M, Hirata Y, Nagai R. Diverse contribution of bone marrow cells to neointimal hyperplasia after mechanical vascular injuries. Circ Res 2003;93:783–790. 14500338 10.1161/01.RES.0000096651.13001.B4

[8] Ross R. The pathogenesis of atherosclerosis: a perspective for the 1990s. Nature 1993;362:801–809. 8479518 10.1038/362801a0

[9] Santos S, Peinado VI, Ramirez J, Melgosa T, Roca J, Rodriguez-Roisin R, et al Characterization of pulmonary vascular remodelling in smokers and patients with mild COPD. Eur Respir J 2002;19:632–638. 11998991 10.1183/09031936.02.00245902

[10] Coll-Bonfill N, de la Cruz-Thea B, Pisano MV, Musri MM. Pflugers Arch. 2016 6;468(6):1071–87 27109570 10.1007/s00424-016-1821-x

[11] Wang Z, Wang DZ, Pipes GC, Olson EN. Myocardin is a master regulator of smooth muscle gene expression. Proc Natl Acad Sci U S A 2003;100:7129–7134. 12756293 10.1073/pnas.1232341100 PMC165841

[12] Fang F, Yang Y, Yuan Z, Gao Y, Zhou J, Chen Q, et al Myocardin-related transcription factor A mediates OxLDL-induced endothelial injury. Circ Res 2011;108:797–807. 10.1161/CIRCRESAHA.111.240655 21330600

[13] Deaton RA, Gan Q, Owens GK. Sp1-dependent activation of KLF4 is required for PDGF-BB-induced phenotypic modulation of smooth muscle. Am J Physiol Heart Circ Physiol 2009;296:H1027–H1037. 10.1152/ajpheart.01230.2008 19168719 PMC2670704

[14] Rieder F, Kessler SP, West GA, Bhilocha S, de la MC, Sadler TM, et al Inflammation-induced endothelial-to-mesenchymal transition: a novel mechanism of intestinal fibrosis. Am J Pathol 2011;179:2660–2673. 10.1016/j.ajpath.2011.07.042 21945322 PMC3204019

[15] Barrallo-Gimeno A, Nieto MA. The Snail genes as inducers of cell movement and survival: implications in development and cancer. Development 2005;132:3151–3161. 15983400 10.1242/dev.01907

[16] Nieto MA. The snail superfamily of zinc-finger transcription factors. Nat Rev Mol Cell Biol 2002;3:155–166. 11994736 10.1038/nrm757

[17] Mistry DS, Chen Y, Wang Y, Zhang K, Sen GL. SNAI2 controls the undifferentiated state of human epidermal progenitor cells. Stem Cells 2014;32:3209–3218. 10.1002/stem.1809 25100569 PMC4339269

[18] Soleimani VD, Yin H, Jahani-Asl A, Ming H, Kockx CE, van Ijcken WF, et al Snail regulates MyoD binding-site occupancy to direct enhancer switching and differentiation-specific transcription in myogenesis. Mol Cell 2012;47:457–468. 10.1016/j.molcel.2012.05.046 22771117 PMC4580277

[19] Torreggiani E, Lisignoli G, Manferdini C, Lambertini E, Penolazzi L, Vecchiatini R, et al Role of Slug transcription factor in human mesenchymal stem cells. J Cell Mol Med 2012;16:740–751. 10.1111/j.1582-4934.2011.01352.x 21645238 PMC3822845

[20] Diez M, Musri MM, Ferrer E, Barbera JA, Peinado VI. Endothelial progenitor cells undergo an endothelial-to-mesenchymal transition-like process mediated by TGFbetaRI. Cardiovasc Res 2010;88:502–511. 10.1093/cvr/cvq236 20631156

[21] Song J, Rolfe BE, Campbell JH, Campbell GR. Changes in three-dimensional architecture of microfilaments in cultured vascular smooth muscle cells during phenotypic modulation. Tissue Cell 1998;30:324–333. 10091337 10.1016/s0040-8166(98)80045-1

[22] Wang X, Hu G, Betts C, Harmon EY, Keller RS, Van De WL, et al Transforming growth factor-beta1-induced transcript 1 protein, a novel marker for smooth muscle contractile phenotype, is regulated by serum response factor/myocardin protein. J Biol Chem 2011;286:41589–41599. 10.1074/jbc.M111.250878 21984848 PMC3308869

[23] Hannus M, Beitzinger M, Engelmann JC, Weickert MT, Spang R, Hannus S, et al siPools: highly complex but accurately defined siRNA pools eliminate off-target effects. Nucleic Acids Res 2014;42:8049–8061. 10.1093/nar/gku480 24875475 PMC4081087

[24] Irizarry RA, Bolstad BM, Collin F, Cope LM, Hobbs B, Speed TP. Summaries of Affymetrix GeneChip probe level data. Nucleic Acids Res 2003;31:e15 12582260 10.1093/nar/gng015 PMC150247

[25] Dai M, Wang P, Boyd AD, Kostov G, Athey B, Jones EG, et al Evolving gene/transcript definitions significantly alter the interpretation of GeneChip data. Nucleic Acids Res 2005;33:e175 16284200 10.1093/nar/gni179 PMC1283542

[26] Smyth GK. Linear models and empirical bayes methods for assessing differential expression in microarray experiments. Stat Appl Genet Mol Biol 2004;3:Article3. 10.2202/1544-6115.1027 16646809

[27] Peinado VI, Barbera JA, Ramirez J, Gomez FP, Roca J, Jover L, et al Endothelial dysfunction in pulmonary arteries of patients with mild COPD. Am J Physiol 1998;274:L908–L913. 9609729 10.1152/ajplung.1998.274.6.L908

[28] Ciuclan L, Bonneau O, Hussey M, Duggan N, Holmes AM, Good R, et al A novel murine model of severe pulmonary arterial hypertension. Am J Respir Crit Care Med. 2011 11 15;184(10):1171–82 10.1164/rccm.201103-0412OC 21868504

[29] Igura T, Kawata S, Miyagawa J, Inui Y, Tamura S, Fukuda K, et al Expression of heparin-binding epidermal growth factor-like growth factor in neointimal cells induced by balloon injury in rat carotid arteries. Arterioscler Thromb Vasc Biol 1996;16:1524–1531. 8977458 10.1161/01.atv.16.12.1524

[30] Scharadin TM, Eckert RL. TIG3: an important regulator of keratinocyte proliferation and survival. J Invest Dermatol 2014;134:1811–1816. 10.1038/jid.2014.79 24599174 PMC4057967

[31] Martinez-Estrada OM, Culleres A, Soriano FX, Peinado H, Bolos V, Martinez FO, et al The transcription factors Slug and Snail act as repressors of Claudin-1 expression in epithelial cells. Biochem J 2006;394:449–457. 16232121 10.1042/BJ20050591 PMC1408675

[32] Jang WG, Kim HS, Park KG, Park YB, Yoon KH, Han SW et al Analysis of proteome and transcriptome of tumor necrosis factor alpha stimulated vascular smooth muscle cells with or without alpha lipoic acid. Proteomics 2004 11 4 (11):3383–9334. 15378733 10.1002/pmic.200400972

[33] Stone MR, O'Neill A, Lovering RM, Strong J, Resneck WG, Reed PW et al Absence of keratin 19 in mice causes skeletal myopathy with mitochondrial and sarcolemmal reorganization. J cell Scie 11 15 120(Pt22):3999–4008). 10.1242/jcs.009241 PMC9202444 17971417

[34] Morales M, Arenas EJ, Urosevic J, Guiu M, Fernández E, Planet E, et al RARRES3 suppresses breast cancer lung metastasis by regulating adhesion and differentiation. EMBO Mol Med. 2014 5 27;6(7):865–81 10.15252/emmm.201303675 24867881 PMC4119352

[35] Zhou C, Nitschke AM, Xiong W, Zhang Q, Tang Y, Bloch M, et al Proteomic analysis of tumor necrosis factor-alpha resistant human breast cancer cells reveals a MEK5/Erk5-mediated epithelial-mesenchymal transition phenotype. Breast Cancer Res 2008;10:R105 10.1186/bcr2210 19087274 PMC2656902

[36] Aomatsu K, Arao T, Abe K, Kodama A, Sugioka K, Matsumoto K, et al Slug is upregulated during wound healing and regulates cellular phenotypes in corneal epithelial cells. Invest Ophthalmol Vis Sci 2012;53:751–756. 10.1167/iovs.11-8222 22247468

[37] Sarrio D, Rodriguez-Pinilla SM, Hardisson D, Cano A, Moreno-Bueno G, Palacios J. Epithelial-mesenchymal transition in breast cancer relates to the basal-like phenotype. Cancer Res 2008;68:989–997. 10.1158/0008-5472.CAN-07-2017 18281472

[38] Cobaleda C, Perez-Caro M, Vicente-Duenas C, Sanchez-Garcia I. Function of the zinc-finger transcription factor SNAI2 in cancer and development. Annu Rev Genet 2007;41:41–61. 17550342 10.1146/annurev.genet.41.110306.130146

[39] Kumar MS, Hendrix JA, Johnson AD, Owens GK. Smooth muscle alpha-actin gene requires two E-boxes for proper expression in vivo and is a target of class I basic helix-loop-helix proteins. Circ Res 2003;92:840–847. 12663487 10.1161/01.RES.0000069031.55281.7C

[40] Qiu P, Li L. Histone acetylation and recruitment of serum responsive factor and CREB-binding protein onto SM22 promoter during SM22 gene expression. Circ Res 2002;90:858–865. 11988486 10.1161/01.res.0000016504.08608.b9

[41] Barath P, Fishbein MC, Cao J, Berenson J, Helfant RH, Forrester JS. Tumor necrosis factor gene expression in human vascular intimal smooth muscle cells detected by in situ hybridization. Am J Pathol 1990;137:503–509. 1698022 PMC1877504

[42] Pitsiou G, Kyriazis G, Hatzizisi O, Argyropoulou P, Mavrofridis E, Patakas D. Tumor necrosis factor-alpha serum levels, weight loss and tissue oxygenation in chronic obstructive pulmonary disease. Respir Med 2002;96:594–598. 12195840 10.1053/rmed.2002.1322

[43] Pizarro S, Garcia-Lucio J, Peinado VI, Tura-Ceide O, Diez M, Blanco I, et al Circulating progenitor cells and vascular dysfunction in chronic obstructive pulmonary disease. PLoS One 2014;9:e106163 10.1371/journal.pone.0106163 25171153 PMC4149524

[44] Takabatake N, Nakamura H, Abe S, Inoue S, Hino T, Saito H, et al The relationship between chronic hypoxemia and activation of the tumor necrosis factor-alpha system in patients with chronic obstructive pulmonary disease. Am J Respir Crit Care Med 2000;161:1179–1184. 10764309 10.1164/ajrccm.161.4.9903022

[45] Ali MS, Starke RM, Jabbour PM, Tjoumakaris SI, Gonzalez LF, Rosenwasser RH, et al TNF-alpha induces phenotypic modulation in cerebral vascular smooth muscle cells: implications for cerebral aneurysm pathology. J Cereb Blood Flow Metab 2013;33:1564–1573. 10.1038/jcbfm.2013.109 23860374 PMC3790924

[46] Storci G, Sansone P, Mari S, D'Uva G, Tavolari S, Guarnieri T, et al TNFalpha up-regulates SLUG via the NF-kappaB/HIF1alpha axis, which imparts breast cancer cells with a stem cell-like phenotype. J Cell Physiol 2010;225:682–691. 10.1002/jcp.22264 20509143 PMC2939957

[47] Shankman LS, Gomez D, Cherepanova OA, Salmon M, Alencar GF, Haskins RM, et al KLF4-dependent phenotypic modulation of smooth muscle cells has a key role in atherosclerotic plaque pathogenesis. Nat Med. 2015 6;21(6):628–37 10.1038/nm.3866 25985364 PMC4552085

[48] Shirley SH, Hudson LG, He J, Kusewitt DF. The skinny on Slug. Mol Carcinog 2010;49:851–861. 10.1002/mc.20674 20721976 PMC3632330

[49] Savagner P, Kusewitt DF, Carver EA, Magnino F, Choi C, Gridley T, et al Developmental transcription factor slug is required for effective re-epithelialization by adult keratinocytes. J Cell Physiol 2005;202:858–866. 15389643 10.1002/jcp.20188

[50] Moreno-Bueno G, Cubillo E, Sarrio D, Peinado H, Rodriguez-Pinilla SM, Villa S, et al Genetic profiling of epithelial cells expressing E-cadherin repressors reveals a distinct role for Snail, Slug, and E47 factors in epithelial-mesenchymal transition. Cancer Res 2006;66:9543–9556. 17018611 10.1158/0008-5472.CAN-06-0479

[51] Savagner P, Yamada KM, Thiery JP. The zinc-finger protein slug causes desmosome dissociation, an initial and necessary step for growth factor-induced epithelial-mesenchymal transition. J Cell Biol 1997;137:1403–1419. 9182671 10.1083/jcb.137.6.1403 PMC2132541

[52] Murray SA, Gridley T. Snail1 gene function during early embryo patterning in mice. Cell Cycle 2006;5:2566–2570. 17106264 10.4161/cc.5.22.3502

[53] Perez-Mancera PA, Gonzalez-Herrero I, Maclean K, Turner AM, Yip MY, Sanchez-Martin M, et al SLUG (SNAI2) overexpression in embryonic development. Cytogenet Genome Res 2006;114:24–29. 16717446 10.1159/000091924

[54] Schultz K, Fanburg BL, Beasley D. Hypoxia and hypoxia-inducible factor-1alpha promote growth factor-induced proliferation of human vascular smooth muscle cells. Am J Physiol Heart Circ Physiol 2006;290:H2528–H2534. 16399861 10.1152/ajpheart.01077.2005

[55] Starke RM, Ali MS, Jabbour PM, Tjoumakaris SI, Gonzalez F, Hasan DM, et al Cigarette smoke modulates vascular smooth muscle phenotype: implications for carotid and cerebrovascular disease. PLoS One 2013;8:e71954 10.1371/journal.pone.0071954 23967268 PMC3743809

[56] Qiao L, Nishimura T, Shi L, Sessions D, Thrasher A, Trudell JR, et al Endothelial fate mapping in mice with pulmonary hypertension. Circulation. 2014 2 11;129(6):692–70 10.1161/CIRCULATIONAHA.113.003734 24201301

