# Correction: Slug Is Increased in Vascular Remodeling and Induces a Smooth Muscle Cell Proliferative Phenotype

**DOI:** 10.1371/journal.pone.0162117

**Published:** 2016-08-25

**Authors:** Núria Coll-Bonfill, Victor I. Peinado, María V. Pisano, Marcelina Párrizas, Isabel Blanco, Maurits Evers, Julia C. Engelmann, Jessica García-Lucio, Olga Tura-Ceide, Gunter Meister, Joan Albert Barberà, Melina M. Musri

In the Funding section, the grant number FIS to VIP is listed incorrectly: The correct grant number to VIP is FIS PI13/00836.

The Funding Section should appear as follows: This work was supported by the Instituto de Salud Carlos III (ISCIII), grants FIS PI13/00836 to VIP and FIS PS09/00536 to JAB, and the Sociedad Española de Pneumologia y Cirugía Toracica (SEPAR), grant SEPAR-2009 to MMM. MMM is recipient of a Sara Borrell contract from ISCIII. JGL is recipient of a Pre-doctoral contract from the ISCIII. MVP is recipient of a Post-doctoral contract from CONICET. This work was also funded by the Fundación Contra la Hipertensión Pulmonar (FCHP). "Cofinanciado por el Fondo Europeo de Desarrollo Regional (FEDER). Unión Europea. Una manera de hacer Europa". The funders had no role in study design, data collection and analysis, decision to publish, or preparation of the manuscript.
