# Supplementary figures and images for: Slug Is Increased in Vascular Remodeling and Induces a Smooth Muscle Cell Proliferative Phenotype
Source: PLoS One. 2016 Jul 21;11(7):e0159460. doi: 10.1371/journal.pone.0159460 (PMC4956159; doi:10.1371/journal.pone.0159460)

**S1 Fig**

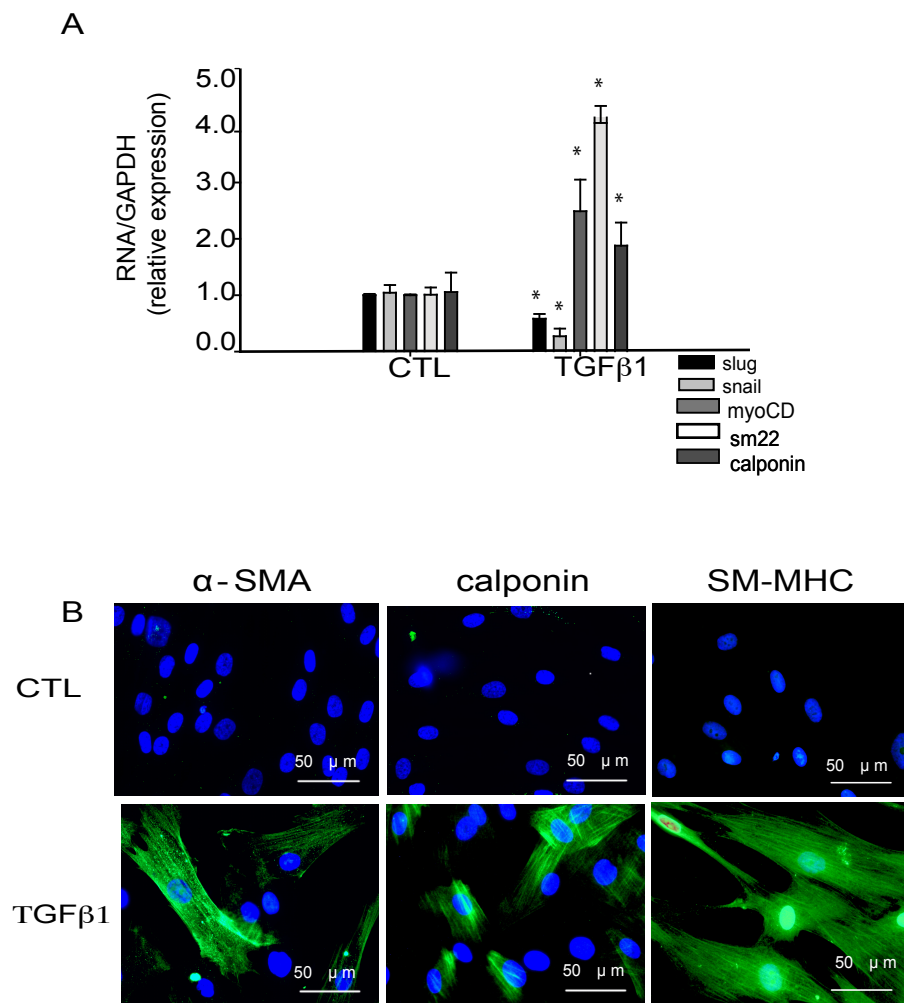

Supplement: S1 Fig — A, RT-PCR and B, immunofluorescence of SMC markers show SMC differentiation after 48 h of TGFβ1 treatment. Slug expression, measured by RT-PCR, decreases in differentiated cells. (PDF) [file pone.0159460.s002.pdf]

S2 Fig

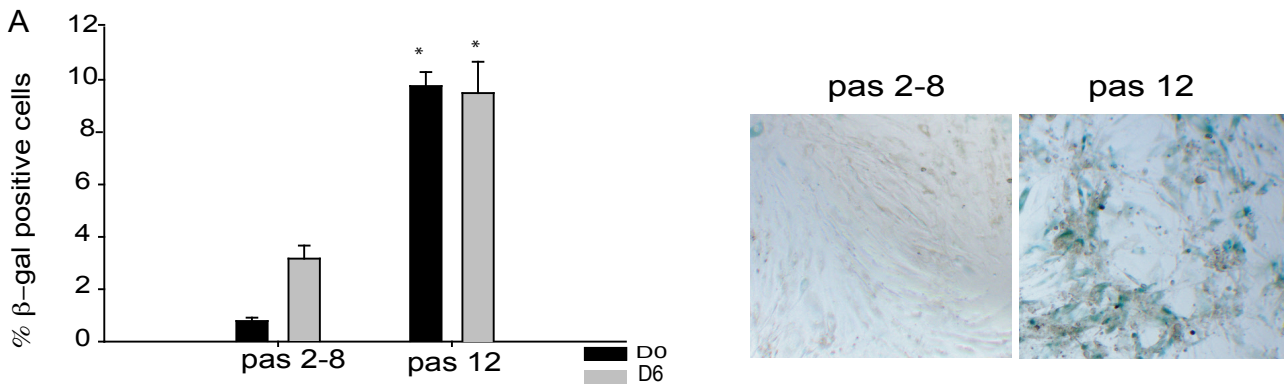

Supplement: S2 Fig — Graph showing the percentage of senescent cells expressed as b-galactosidase positive cells/total cells. No differences are observed between D0 and D6 (passage 2–8). Passage 12 is used as a control for cell senescence. *p < 0.05 by one-way ANOVA (PDF) [file pone.0159460.s003.pdf]

**S3 Fig**

sictl

sislug

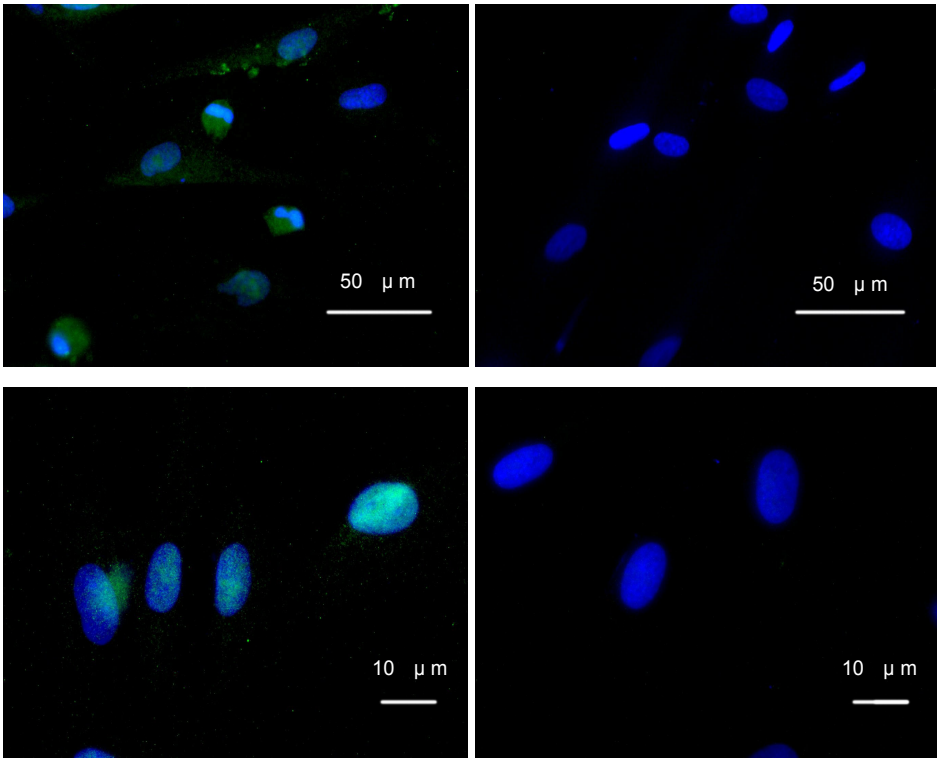

Supplement: S3 Fig — Representative Slug immunofluorescence, which demonstrates the decrease of Slug expression around 90% with respect to control cells. (PDF) [file pone.0159460.s004.pdf]

S4 Fig

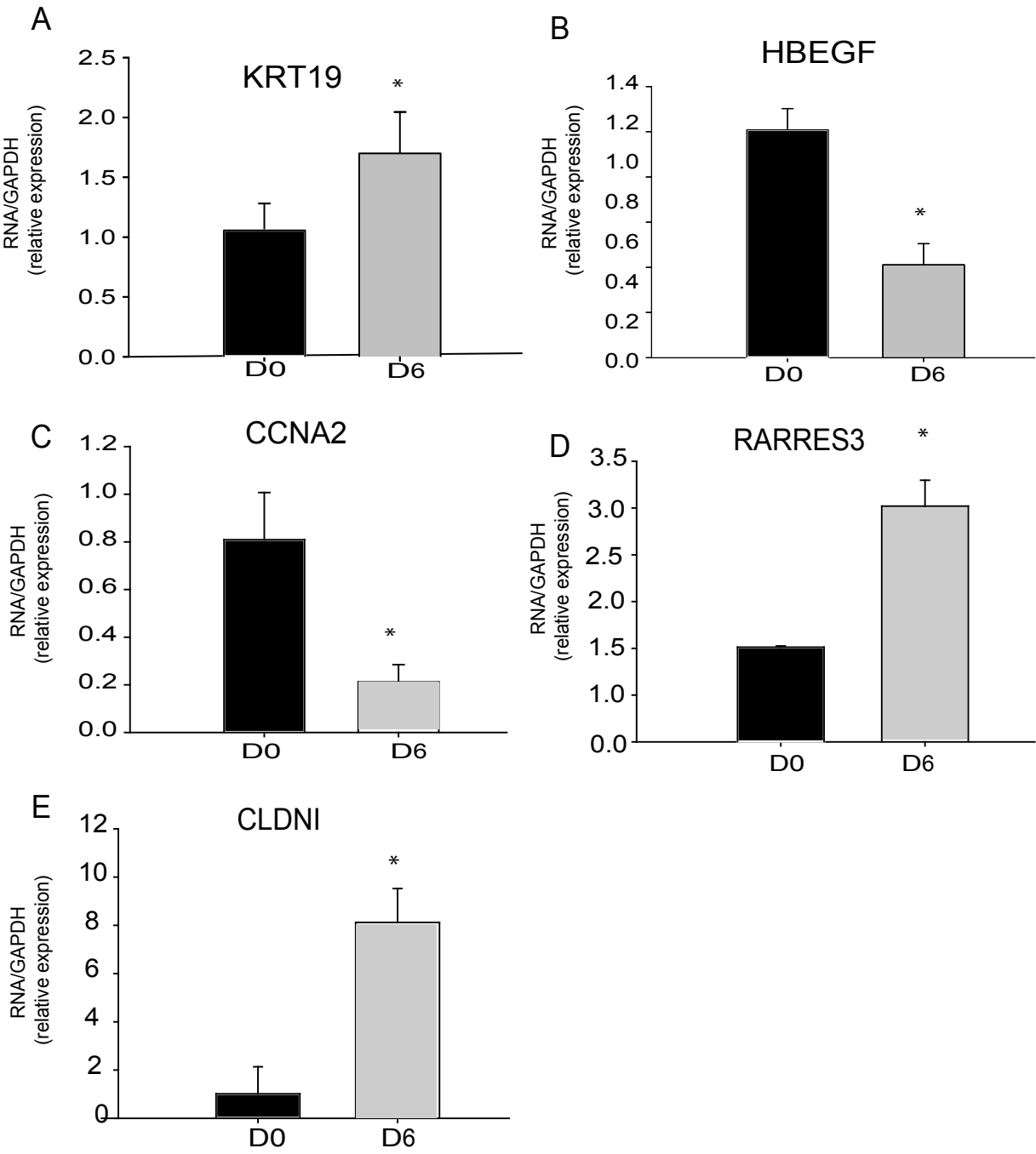

Supplement: S4 Fig — KRT19 (A), RARRES3 (D) and CLDNI (E) increase in differentiated SMC, whereas CCNA2 (C) and HBGEF (B) decrease. (PDF) [file pone.0159460.s005.pdf]

**S5 Fig**

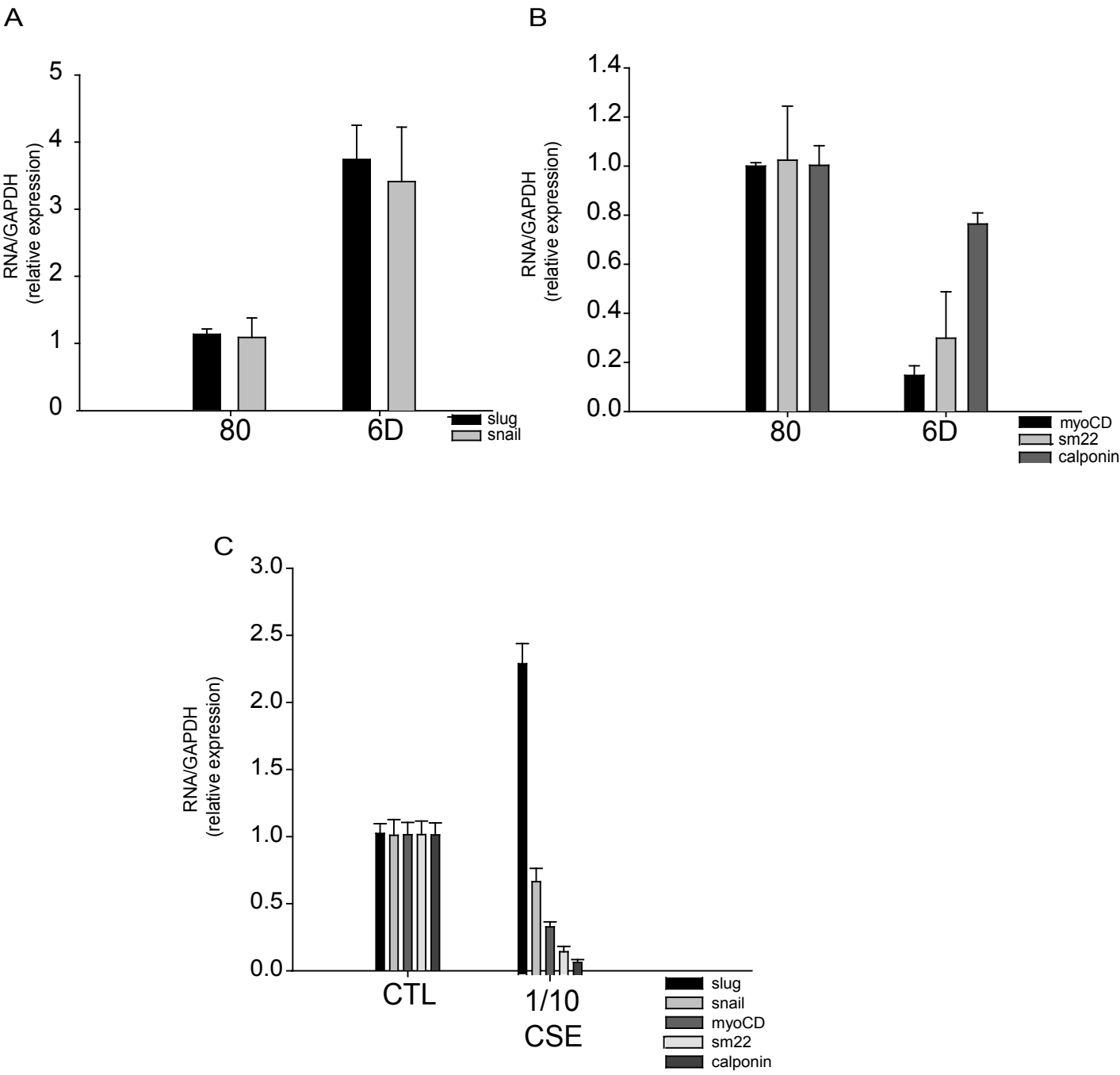

Supplement: S5 Fig — A-B Slug expression increases in SMC, correlating with SMC dedifferentiation, as shown by the downregulation of SMC marker genes, in hypoxia-induced cells. C, CSE stimulation promotes the increase of Slug expression and the decrease of SMC differentiation markers. Data are expressed as the mean ± SEM of at least three independent experiments performed in duplicate. *p < 0.05 by paired t-test. (PDF) [file pone.0159460.s006.pdf]
