# Supplementary material for: Slug Is Increased in Vascular Remodeling and Induces a Smooth Muscle Cell Proliferative Phenotype
Source: PLoS One. 2016 Jul 21;11(7):e0159460. doi: 10.1371/journal.pone.0159460 (PMC4956159; doi:10.1371/journal.pone.0159460)
Supplement: S1 Table — (PDF) [file pone.0159460.s007.pdf]

Gene	Sequence
Calponin F	CACGACATTTTTGAGGCCAA
Calponin R	TTTCCTTTCGTCTTCGCCAT
Sm22v2 F	GGAAGCCTTCTTTCCCCAGA
Sm22v2 R	TCCAGCTCCTCGTCATACTTCTT
MyoCD F	GCACCAAGCTCAGCTTAAGGA
MyoCD R	TGGGAGTGGGCCTGGTTT
Slug F	GCAAGATCTGCGGCAAGG
Slug R	GCTCTGTTGCAGTGAGGGC
Snail F	CCCAGTGCCTCGACCACTAT
Snail R	CCAGATGAGCATTGGCAGC
VE- caherina F	GATGCAGACGACCCCACTGT
VE- cadherina R	CCACGATCTCATACCTGGCC
CD31 F	AAAGTCGGACAGTGGGACGT
CD31 R	GGCTGGGAGAGCATTTCACA
GAPDH F	CATCACCATCTTCCAGGAGC
GAPDH R	TGGACTCCACGACGTACTCA
βactin F	CGGAACGCCTCATTGCC
βactin R	ACCCACACTGTGCCCATCTA
Ki67 F	GCAGCCTTAACTGTGACACTTGC
Ki67 R	GCCACCGTGCCCTGG
KLF4 F	GGGAGAAGACACTGCGTCA
KLF4 R	GGAAGCACTGGGGGAAGT
Caldesmon F	GGAGGTGAATGCCCAGAACA
Caldesmon R	AGGAATGCGGCCTCATCATC
Gp βactin F	ATATCGCTGCGCTCGTTGTC
Gp βactin R	AACGATGCCGTGCTCAATG
Gp slug F	AGACCCTGGTTGCTTCAAGGA
Gp slug R	GTTGCAGTGCGGGCAAG
Gp snail F	CTTACCTTCCAGCAGCCCTA
Gp snail R	GGAGTCCCAGATGAGTGTCG
Mouse βactin F	GGAGGGGGTTGAGGTGTT
Mouse βactin R	GTGTGCACTTTTATTGGTCTCAA
Mouse slug F	TGTATGGACATCGTCGGCAG
Mouse slug R	ACTTACACGCCCCAAGGATG
Mouse snail F	TGGAAAGGCCTTCTCTAGGC
Mouse snail R	AAAGCACGGTTGCAGTGG
TIG3 F	TCTGGCTCCTCCAAGTGAGT
TIG3 R	TTTCACCTCTGCACTGTTGC
CLDNI F	CGATGAGGTGCAGAAGATGA
CLDNI R	AGCCAGACCTGCAAGAAGAA
GATA 6F	TTCCCATGACTCCAACTTCC
GATA 6 R	TGGGGGAAGTATTTTTGCTG
CCNA2 F	CCATACCTCAAGTATTTGCCATC
CCNA2 R	TCCAGTCTTTCGTATTAATGATTCAG
HBEGF F	GGACCAGCTGCTACCCCTA
HBEGF R	GTGGCTTGGAGGATAAAGTGA

Supplementary Table I . Summary of Primers used in this study
