# Supplementary material for: Slug Is Increased in Vascular Remodeling and Induces a Smooth Muscle Cell Proliferative Phenotype
Source: PLoS One. 2016 Jul 21;11(7):e0159460. doi: 10.1371/journal.pone.0159460 (PMC4956159; doi:10.1371/journal.pone.0159460)
Supplement: S2 Table — (PDF) [file pone.0159460.s008.pdf]

Cell cycle (GO:0007049)	
Gene symbol	Rank metric score
MAD2L1	-0.406027645
NEK2	-0.391486853
BIRC5	-0.360862374
TTK	-0.345425099
DLGAP5	-0.340312243
KIF23	-0.330975831
NDC80	-0.318150222
CENPF	-0.306908071
BUB1B	-0.303575546
CENPE	-0.300727546
UBE2C	-0.299725682
BUB1	-0.285587072
MLF1	-0.285531104
KIF11	-0.283260554
PRC1	-0.259448439
CIT	-0.256977081
CDC6	-0.253449202
ZWINT	-0.252591521
ANLN	-0.252140313
KIF15	-0.247360334
RAD51	-0.246333823
AFAP1L2	-0.245889321
PRUNE2	-0.238055795
DIRAS3	-0.237249017
PLK1	-0.234444648
KIF2C	-0.226172
NCAPH	-0.225722983
NUSAP1	-0.22515744
E2F1	-0.218571082
CDC45	-0.215077609

Supplementary Table II
