# Supplementary material for: Slug Is Increased in Vascular Remodeling and Induces a Smooth Muscle Cell Proliferative Phenotype
Source: PLoS One. 2016 Jul 21;11(7):e0159460. doi: 10.1371/journal.pone.0159460 (PMC4956159; doi:10.1371/journal.pone.0159460)
Supplement: S3 Table — (PDF) [file pone.0159460.s009.pdf]

Term	Size	OddsRatio	Pvalue	GOBPID
cellular response to stimulus	4889	2,274211678	0,008233	GO:0051716
cell communication	4616	2,240660138	0,009362	GO:0007154
response to stress	2883	3,50146648	0,000142	GO:0006950
regulation of response to stimulus	2413	2,872706422	0,002001	GO:0048583
response to organic substance	1942	3,727620654	0,000189	GO:0010033
cell proliferation	1498	2,765109381	0,008739	GO:0008283
regulation of programmed cell death	1048	3,071960298	0,009225	GO:0043067
positive regulation of signal transduction	819	3,396551724	0,00838	GO:0009967
immune response	730	7,299385774	2,55E-05	GO:0006955
interspecies interaction between organisms	640	4,418888231	0,002165	GO:0044419
multi-organism cellular process	587	4,842672414	0,001319	GO:0044764
inflammatory response	478	4,987673344	0,002343	GO:0006954
regulation of defense response	438	5,466329966	0,001505	GO:0031347
regulation of cell motility	415	4,666427547	0,006607	GO:2000145
modification of morphology	350	5,573316283	0,003218	GO:0051817

Supplemental Table III : Summary of GO Terms analysis of Biological Pathways.
