# Supplementary material for: Slug Is Increased in Vascular Remodeling and Induces a Smooth Muscle Cell Proliferative Phenotype
Source: PLoS One. 2016 Jul 21;11(7):e0159460. doi: 10.1371/journal.pone.0159460 (PMC4956159; doi:10.1371/journal.pone.0159460)
Supplement: S4 Table — * p<0.005 vs NS. † p<0.005 vs S. (PDF) [file pone.0159460.s010.pdf]

	NS (n=4)	S (n=7)	COPD (n=7)
**BMI (kg/m^2)**	25 ± 5	27± 4	26± 3
**FEV1 (%predicted)**	102 ± 15	90± 13	67± 11* †
**FVC (%predicted)**	110± 26	119± 13	99± 19
**FEV1/FVC (%predicted)**	73± 5	74± 4	57± 9*†
**Dlco (%predicted)**	92± 9	83± 8	71± 10*
**Pa02 (mmHg)**	90± 19	87± 11	75± 9*
**Distribution R1\R2\R3**	1\1\2	2\3\2	1\3\3
